# Inflammation‐Responsive Hydrogel Spray for Synergistic Prevention of Traumatic Heterotopic Ossification via Dual‐Homeostatic Modulation Strategy

**DOI:** 10.1002/advs.202302905

**Published:** 2023-08-27

**Authors:** Jiazhao Yang, Xudong Zhang, Baoliang Lu, Jiawei Mei, Lei Xu, Xianzuo Zhang, Zheng Su, Wei Xu, Shiyuan Fang, Chen Zhu, Dongdong Xu, Wanbo Zhu

**Affiliations:** ^1^ Department of Orthopedics The First Affiliated Hospital of USTC University of Science and Technology of China Hefei Anhui 230001 P. R. China; ^2^ Department of Orthopedics Shanghai Jiao Tong University Affiliated Sixth People's Hospital Shanghai Jiao Tong University Shanghai 200233 P. R. China

**Keywords:** dual‐homeostatic modulation, efferocytosis, hydrogel spray, inflammation‐responsive, traumatic heterotopic ossification

## Abstract

Traumatic heterotopic ossification (THO) represents one of the most prominent contributors to post‐traumatic joint dysfunction, which currently lacks an effective and definitive preventative approach. Inflammatory activation due to immune dyshomeostasis during the early stages of trauma is believed to be critical in initiating the THO disease process. This study proposes a dual‐homeostatic modulation (DHM) strategy to synergistically prevent THO without compromising normal trauma repair by maintaining immune homeostasis and inducing stem cell homeostasis. A methacrylate‐hyaluronic acid‐based hydrogel spray device encapsulating a curcumin‐loaded zeolitic imidazolate framework‐8@ceric oxide (ZIF‐8@CeO2, CZC) nanoparticles (CZCH) is designed. Photo‐crosslinked CZCH is used to form hydrogel films fleetly in periosteal soft tissues to achieve sustained curcumin and CeO2 nanoparticles release in response to acidity and reactive oxygen species (ROS) in the inflammatory microenvironment. In vitro experiments and RNA‐seq results demonstrated that CZCH achieved dual‐homeostatic regulation of inflammatory macrophages and stem cells through immune repolarization and enhanced efferocytosis, maintaining immune cell homeostasis and normal differentiation. These findings of the DHM strategy are also validated by establishing THO mice and rat models. In conclusion, the CZCH hydrogel spray developed based on the DHM strategy enables synergistic THO prevention, providing a reference for a standard procedure of clinical operations.

## Graphical abstract

This study proposes a dual‐homeostatic modulation (DHM) strategy to synergistically prevent THO without compromising normal trauma repair by maintaining immune homeostasis and inducing stem cell homeostasis. A methacrylate‐hyaluronic acid‐based hydrogel spray device encapsulating a curcumin‐loaded zeolitic imidazolate framework‐8@ceric oxide nanoparticles (CZCH) is designed. CZCH achieved dual‐homeostatic regulation of inflammatory macrophages and stem cells through immune repolarization and enhanced efferocytosis, maintaining immune cell homeostasis and normal differentiation. These findings of the DHM strategy are also validated by establishing THO mice and rat models.

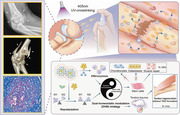

## 1 Introduction

Traumatic heterotopic ossification (THO) refers to the post‐traumatic formation of bone tissue within soft tissues such as muscles, tendons, and ligaments, commonly around major joints such as the hip and elbow.^[^ ^1^ ^]^ It usually disrupts the original morphological structure of the soft tissues, exacerbates painful symptoms, reduces joint mobility, and ultimately leads to joint stiffness and loss of function.^[^ ^2^ ^]^ The lack of consensus on the pathogenesis, elusive sites of onset, and lack of preventive measures have largely limited the routine prevention of THO.^[^ ^3^ ^]^ It is widely thought that the endochondral osteogenic pathway accounts predominantly for the pathogenesis of THO, consists of four phases: inflammation, chondrogenesis, osteogenesis, and bone maturation.^[^ ^4^ ^]^ After activation of the inflammatory cascade, inflammatory immune cells such as macrophages chemotactic infiltrate and recruit stem cells through the vasculature, leading to fibroblast recruitment, proliferation, and fibrosis.^[^ ^5^ ^]^ The current primary THO prevention approach, the intake of non‐steroidal anti‐inflammatory drugs (NSAIDs), aims to prevent THO by mitigating the initial inflammatory activation of THO.^[^ ^6^ ^]^ However, the limited effect of NSAIDs in a local hypoxic environment and the side effects restrict their broad application.^[^ ^7^ ^]^ Over the years, several treatments focused on inhibiting the osteogenic process, including administering bone morphogenetic protein 2 (BMP2) inhibitors and osteoclast inducers.^[^ ^8^ ^]^ However, these strategies also have implications for normal bone and tissue healing.^[^ ^9^ ^]^ Extensive surgical excision remains the mainstay of treatment.^[^ ^10^ ^]^

Abnormal persistent inflammatory activation of macrophages in the post‐traumatic immune microenvironment, which promotes abnormal osteogenic differentiation of stem cells, is the most important stage in the pathological process of THO.^[^ ^11^ ^]^ The macrophage phenotype plays a complex dual role in the progression of THO.^[^ ^12^ ^]^ M1 macrophages, with a pro‐inflammatory role, initiate inflammatory infiltration and recruitment, whereas M2 macrophages, with a tissue repair‐promoting role, are responsible for the osteogenic differentiation of stem cells.^[^ ^13^ ^]^ In the specific immune microenvironment of THO, the M1/M2 balance of the macrophage immunophenotype is disrupted, and the impaired macrophage function may be the most important reason for the eventual formation of THO^[^ ^14^ ^]^ The traumatic factors leading to macrophage dyshomeostasis originate from the hypoxic microenvironment, the increased reactive oxygen species (ROS) due to oxidative stress, and the cascade release of inflammatory factors.^[^ ^15^ ^]^ Curcumin, a natural drug derived from the rhizomes of plants of the Ginger families, has been documented to modulate M1/M2 homeostasis in immune macrophages.^[^ ^16^ ^]^ In an immune microenvironment with abnormal inflammatory activation, curcumin inhibits the production of pro‐inflammatory cytokines and chemokines by M1 macrophages, thereby suppressing their induced activation of metalloproteinases, adhesion molecules and pro‐angiogenic factors, as well as signaling pathways such as NF‐κB, mTOR and STATs.^[^ ^17^ ^]^ However, no direct studies have demonstrated that curcumin can inhibit THO formation.

The local tissue confinement of trauma may limit the effectiveness of systemic drug delivery, while the difficulty in defining the extent of THO onset limits the application of local drug delivery.^[^ ^18^ ^]^ Sprayable hydrogel is a topical drug delivery measure that uses devices to spray liquid hydrogel into the trauma and crosslink in situ under external conditions to form a hydrogel film.^[^ ^19^ ^]^ Hydrogel sprays offer the advantages of portability and rapid in situ action, with the ability to spray rapidly to create close contact with surrounding tissue and provide sufficient flexibility to spray for wide drug delivery.^[^ ^20^ ^]^ Hyaluronic acid (HA) is a naturally occurring unbranched polysaccharide that is a major component of the extracellular matrix (ECM) with superior biocompatibility and biodegradability. The methacrylate‐catalyzed HA (HAMA) possesses rapid UV cross‐linking properties that allow for rapid cross‐curing within seconds, permitting its direct use as an intraoperative concomitant THO preventive measure.

**Scheme 1:**
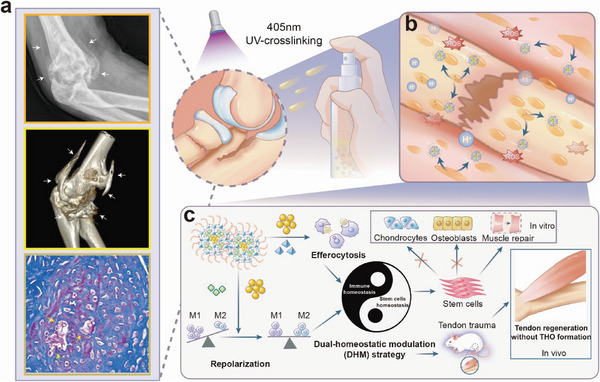
Schematic design of CZCH hydrogel sprayer based on DHM strategy. a) THO formation around elbow trauma (X‐ray and CT 3D reconstructions, white arrows) and around Achilles tendon trauma in rats (Mason staining, yellow arrows). b) CZCH with inflammatory responsive release properties accelerates the release of CZC within the acidic and H_2_O_2_‐rich trauma microenvironment. c) The CZC released in CZCH maintained immune homeostasis through immune repolarization and pro‐efferocytosis effects, and further enhanced the regulation of stem cell homeostasis, avoiding mis‐differentiation of stem cells to chondrogenic and osteogenic cells.

For the specific immune microenvironment of THO, we proposed a dual‐homeostatic modulation (DHM) strategy for simultaneously maintaining the immune homeostasis of THO‐associated macrophages and stem cells. Based on the characteristics of acidity and high ROS content in the inflammatory microenvironment of trauma, we designed an acid‐responsive zeolitic imidazolate framework‐8 encapsulating curcumin (Cur@ZIF‐8) and further encapsulated Cur@ZIF‐8 using ROS‐responsive cerium oxide nanoparticles (CeO_2_ NPs) and polyvinyl pyrrolidone (PVP) to form Cur@ZIF‐8@CeO_2_ (CZC) nano‐MOF.^[^ ^21^ ^]^ Using ultrasonic shaking, CZC nano‐MOF was dispersed in HAMA hydrogel solution to prepare an inflammation‐responsive hydrogel spray CZCH with DHM function. In vitro experiments revealed that the CZCH spray could stably form and cross‐link dense hydrogel films with enhanced immune homeostasis modulation and tissue repair ability. In dual THO animal models in rats and mice, the CZCH hydrogel spray successfully implemented the DHM strategy to prevent THO formation and promote normal healing of traumatized tendons. These results validated the early and comprehensive preventive effect of CZCH hydrogel spray on THO, providing the theoretical basis for clinical application and commercial translation (**Scheme** **1**).

## 2 Results and Discussion

### 2.1 Characterization of CZC

**Figure 1:**
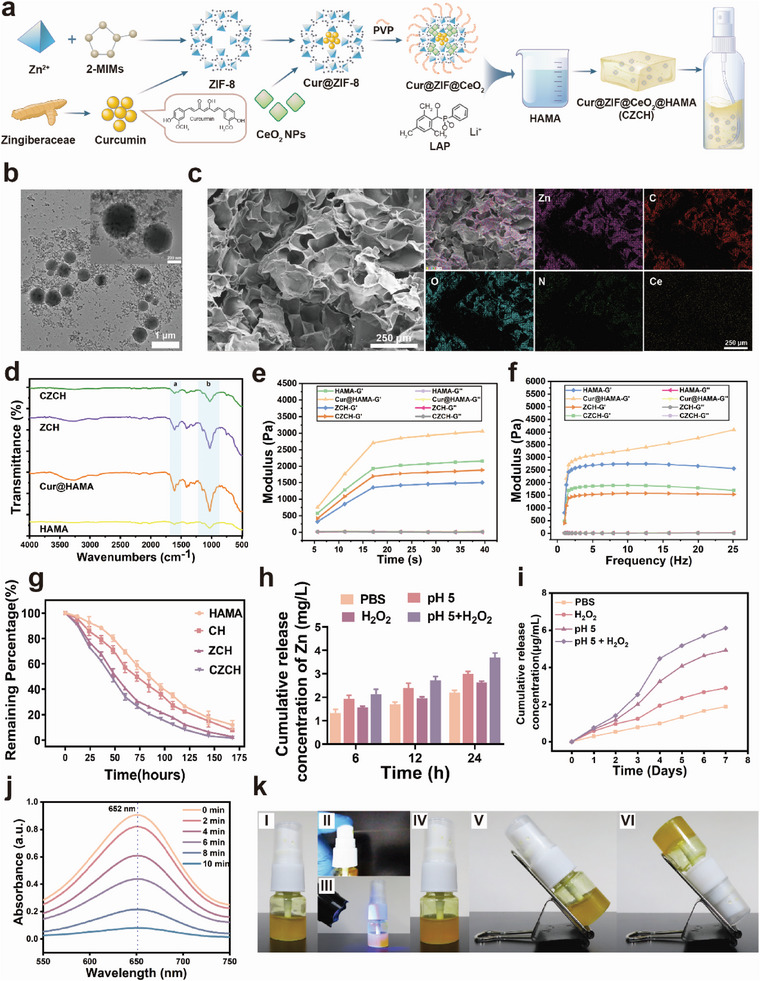
Characterization of CZCH. a) Schematic diagram of the synthesis process of CZCH. b) TEM image of CZC. Scale bar, 1 µm. The embedded image is a local magnification. scale bar, 200 nm. c) SEM image of lyophilized cross‐section and elemental mapping of CZCH hydrogel. Scale bar, 250 µm. d) Fourier transform infrared (FTIR) spectra of HAMA, Cur@HAMA, ZCH, and CZCH hydrogels. a and b regions are the characteristic absorption peaks of curcumin. e) Dynamic time scan and f) dynamic frequency scan of the four groups of hydrogels measured by rotational rheometer. g) Degradation curves of the four groups of hydrogels in acidic PBS (pH 5). h) Release of elemental Zn from CZCH in neutral PBS, acidic PBS (pH 5), neutral PBS containing 50 mm H2O2 and acidic PBS containing 50 mm H2O2 as determined by ICP‐MS. i) Cumulative concentration of curcumin released from CZCH in the aforementioned conditions. j) Degradation curve of CZCH in MB solution as determined by UV–vis spectrum. k) CZCH morphology and photo crosslinking ability: I) CZCH before crosslinking, II) CZCH with spraying ability, III) CZCH in rapid photo crosslinking, IV) CZCH after crosslinking, V) CZCH in oblique, and VI) CZCH in inverted without changing morphology.

A schematic diagram of the synthesis process of CZC and CZCH was shown in **Figure** **1**a. The morphology of CZC was observed by transmission electron microscopy (TEM) (Figure 1b; Figure S1a, Supporting Information), exhibiting a regular circular shape with a particle size of about 300 nm and uniform dispersion. EDS mapping revealed 16.35% Zn and 1.37% Ce in CZC, validating the successful synthesis of the materials (Figure S1b, Supporting Information). As shown in Figure S2a (Supporting Information), the average particle size of CZC by Dynamic Light Scattering (DLS) was 323 nm, indicating that the synthesized CZC is of good purity and relatively homogeneous in structure and size. Compared with the particle size of ZIF‐8 MOF reported in the literature, the particle size of CZC was slightly increased, which may be related to the adsorption of CeO_2_ nanoparticles and the modification of PVP.^[^ ^22^ ^]^ As shown in Figure S2b (Supporting Information), the zeta potentials of unloaded ZIF‐8@CeO_2_ and loaded CZC were ≈−30, validating their good dispersion. The synthesis of CZC was further validated using Fourier transform infrared (FTIR) analysis (Figure S3, Supporting Information). The ‐OH stretching vibration at 3250–3500 cm^−1^ corresponded to hydrogen bond formation (especially intramolecular hydrogen bonds) that increased and broadened the absorption intensity. Although the main absorption peaks of curcumin (1634, 1602, 1506, 1026, 960, 855 cm^−1^) were observed in the spectrum, their intensity was changed, indicating that curcumin is bound inside the ZIF‐8@CeO_2_ carrier molecule. X‐ray diffraction (XRD) analysis was conducted to demonstrate the typical ZIF‐8 crystal conformation of CZC (Figure S4a, Supporting Information). The peaks located at 7.3, 12.7, 18.0, and 26.7 corresponded to the (011), (112), (222), and (134) crystallographic planes of ZIF‐8, respectively.^[^ ^23^ ^]^ X‐ray photoelectron spectroscopy (XPS) analysis verified that Zn and Ce elements were present in CZC and their valence states, confirming the successful synthesis of CZC (Figure S4b, Supporting Information).

### 2.2 Characterization of CZCH

CZCH was vacuum lyophilized, then the cross‐sectional morphology and elemental distribution were observed using scanning electron microscopy. As shown in Figure 1c and Figure S5 (Supporting Information), the cross‐section of CZCH exhibited a porous and uniformly dispersed structure with a pore diameter of ≈80 µm and a porosity of about 85%. EDS mapping showed the uniform size of Zn and Ce elements, suggesting a uniform dispersion of CZC in the hydrogel. The FT‐IR assay of CZCH verified the successful synthesis of CZCH (Figure 1d). Due to the large amount of carbon‐carbon double bonds in HAMA and the addition of photoinitiator LAP in the hydrogel precursor solution, the free radical polymerization of carbon‐carbon double bonds occurred under the irradiation of blue light at 405 nm, forming a cross‐linked network and rapidly curing into a hydrogel. Therefore, a rheological analyzer was used to investigate the effect of the rheological properties of the hydrogel before and after cross‐linking of CZCH. As shown in Figure 1e, a dynamic time scan was first performed. We found that the hydrogel modulus of each group did not change significantly with time after experiencing a rapid rise within 20 s after blue light irradiation. The storage modulus *G*' was significantly larger than *G*″, indicating the complete formation of the hydrogel. In addition, the storage modulus G' of CZCH was already close to 500 Pa at the first recording point at 5 s, and G' reached 1700 Pa after curing, indicating rapid light curing performance, which allows the CZCH spray to form hydrogel films in situ quickly during surgery, promoting a broad and sustained controlled release of the drug from CZC. The linear viscoelastic zone of each group of hydrogels revealed by dynamic frequency scanning is shown in Figure 1f. The linear viscoelastic region of each group of hydrogels after photo‐crosslinking was larger than before crosslinking, suggesting that the stability of the hydrogels was further enhanced after photo‐crosslinking.

The swelling and degradation properties of CZCH hydrogels were evaluated for their effect on drug release. The swelling of CZCH in phosphate buffer saline (PBS) solution was first observed, as shown in Figure S6 (Supporting Information). There was no significant difference in the swelling rate among the four groups of hydrogels, which plateaued after 20 h, indicating maximum hydrogel swelling. HAMA hydrogel's ability to absorb and clear exudate due to its excellent swelling properties can help prevent tissue edema and inflammatory infiltration during the early stages of trauma. Given that the degradation of hydrogels is closely related to the controlled release of drugs, we studied the degradation properties of each group of hydrogels in PBS at neutral pH, PBS at acidic pH (pH 5), and solutions with 10% fetal bovine serum (FBS) added. As shown in Figure S7 (Supporting Information), the degradation rate of each group of hydrogels in neutral PBS solution and physiologically simulated environment with the addition of serum was comparable. In contrast, CZCH and ZCH yielded a faster degradation rate in an acidic solution, which may be related to the instability of the ZIF‐8 structure in acidic media (Figure 1g). The presence of surface oxygen vacancies and mutually variable valence states of Ce^3+^ and Ce^4+^ on the CeO_2_ surface can mimic superoxide dismutase (SOD) and catalase (CAT) activities to scavenge ROS, including H_2_O_2_. In the presence of hydrogen peroxide, the gating layer formed by using PVP‐modified CeO_2_ could accelerate the reaction and disintegration, facilitating the release of the drug. Therefore, we investigated the ion release rates of CZCH in different environments using inductively coupled plasma mass spectrometry (ICP‐MS). As shown in Figure 1h and Figure S8 (Supporting Information), CZCN exhibited faster Zn and Ce release in acidic solution (pH = 5), hydrogen peroxide solution (H_2_O_2_, 1 m) and acidic solution with H_2_O_2_. The drug release profile of curcumin investigated using a UV spectrophotometer (UV–vis) revealed that CZCH yielded faster drug release of curcumin (Figure 1i; Figure S9, Supporting Information). Given that curcumin is an excellent antioxidant, we investigated the ability of CZCH to scavenge ROS. As shown in Figure 1j, CZCH significantly reduced the characteristic peaks of hydroxyl radicals (1,3,5‐trimethylbenzene (TMB) method) and scavenged the H_2_O_2_ content in the solution (colorimetric method, Figure S10, Supporting Information). Subsequently, we preliminarily assessed the application of CZCH. As shown in Figure 1k, The CZCH in the spraying device possesses favorable spraying performance and not change its morphology with the movement of the bottle after rapid photo‐crosslinking, which proves the completion of photo‐crosslinking. CZCH sprayed by the spray device could form a broad aqueous mist before photo‐curing. After rapid photo‐crosslinking with 405 nm blue light, CZCH formed an adhesive hydrogel film layer on the glove surface and metal surface without deformation under inclined and inverted positions, as well as under gripping and vertical movements (Figures S11 and S12, Supporting Information). In summary, CZCH exhibits the release of curcumin in response to acidic stimuli and ROS in an inflammatory microenvironment and scavenges excess ROS. These properties make CZCH suitable for application in THO prevention.

### 2.3 CZCH‐Modulated Immune Homeostasis

Biosafety testing was first performed prior to CZCH biological applications. As shown in Figure S13 (Supporting Information), the cell counting kit‐8 (CCK‐8) assay suggested that the relative cells viability of CZC was below 80% at concentrations above 300 µg mL^−1^, while it was above 90% at concentrations up to 100 µg mL^−1^. The hemolysis assay and its quantification further revealed slight hemolysis at concentrations above 200 µg mL^−1^, while no significant hemolysis was observed in the saline group compared to the 100 µg mL^−1^ concentration of CZC (Figure S14, Supporting Information). Therefore, a concentration of 100 µg mL^−1^ of CZC in CZCH was used for the subsequent biological experiments.

**Figure 2:**
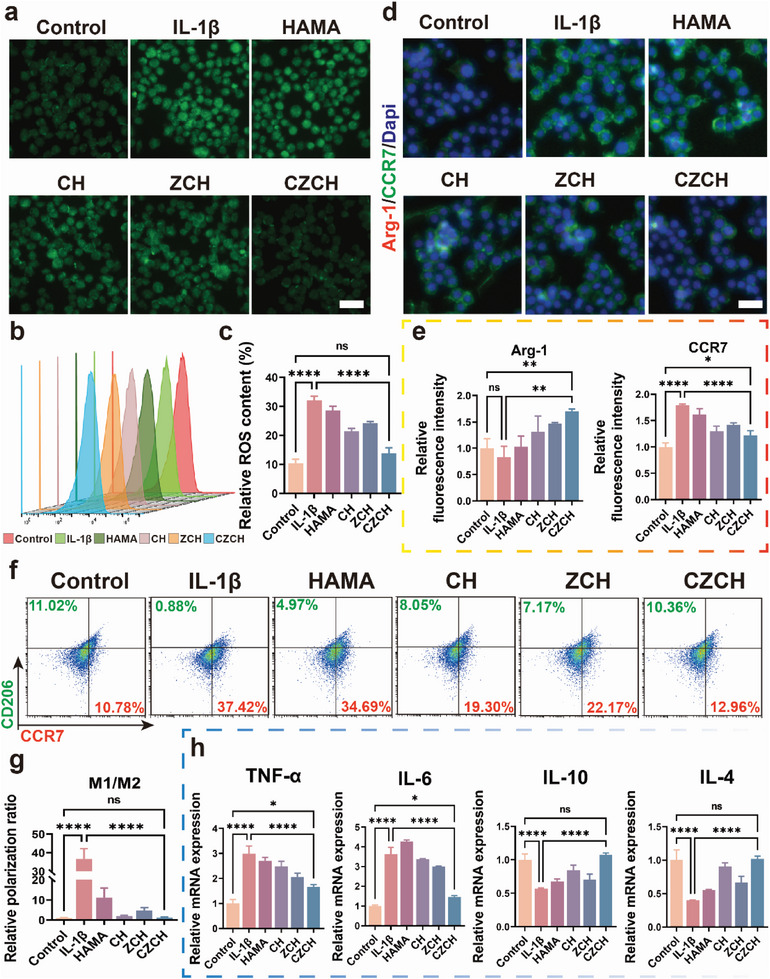
Modulation on immune repolarization of macrophages by CZCH in vitro. a) Intracellular ROS fluorescence images of macrophages after co‐incubation by blank group (Control), IL‐1β group, IL‐1β + HAMA group, IL‐1β + CH group, IL‐1β + ZCH group, and IL‐1β + CZCH group. Scale bar, 50 µm. b) Flow cytometric histogram of intracellular ROS in macrophages after co‐incubation by each group and c) corresponding relative ROS quantification (%). d) Immunofluorescence images of macrophage Arg‐1 (red) and CCR7 (green) after co‐incubation with each group and e) corresponding relative fluorescence intensity quantification. Scale bar, 50 µm. f) Flow cytometry of macrophages CCR7 (M1) and CD206 (M2) after co‐incubation with each group and g) quantification of the corresponding percentage of positivity. h) Cytokine concentrations of TNF‐α, IL‐6, IL‐10, and IL‐4 in macrophage culture medium after co‐incubation with each group as determined by ELISA assay. *p<0.05, **p<0.01, ***p<0.001, ****p<0.0001, ns means no significance.

To better elucidate the role of each component of CZCH, we set up a blank group (Control group), IL‐1β group (supplementation of IL‐1β to the medium to mimic trauma‐induced inflammatory microenvironment) and HAMA group (supplementation of IL‐1β along with the corresponding extracts from HAMA), Cur@HAMA (CH group), ZIF‐8@CeO_2_@HAMA (ZCH group) and CZCH group. We initially explored the role of CZCH in regulating immune cell homeostasis in the inflammatory microenvironment, where immune cells overexpress intracellular ROS due to inflammatory activation, and ROS accumulation leads to the sustained release of pro‐inflammatory factors. In the THO microenvironment, the continuous production of ROS by immune cells can promote dysregulation of stem cell redox homeostasis, leading to the differentiation of stem cells in the wrong direction. As shown in **Figure** **2**a, the intracellular ROS content of macrophages after each treatment was detected using a DCFH‐DA fluorescent probe and characterized using fluorescence imaging. IL‐1β induced significant intracellular ROS accumulation in macrophages compared to Controls. In contrast, intracellular ROS decreased significantly after treatment in the CH, ZCH and CZCH groups. Both curcumin and CeO_2_ nanoparticles yielded anti‐oxidative stress ability, and flow cytometry also verified the most significant ROS scavenging properties of CZCH (Figure 2b,c). The most striking feature of immune dysregulation in THO is the abnormally persistent infiltration of immune cells induced by the cytokine storm during the initial stages of trauma. Inhibition of excessive inflammatory activation of immune cells and restoration of normal immune cell subpopulation ratios represents a potential way to regulate immune homeostasis. To investigate the regulatory effect of CZCH on macrophage homeostasis, we observed the expression of M1 marker CCR7 and M2 marker Arg‐1 by immunofluorescence (IF) staining. Semi‐quantitative fluorescence analysis revealed that the ratio of macrophages treated with CZCH was close to that of the Control group (Figure 2d,e). Although inflammatory activation is the initiating triggering factor for THO, the excessive release of TGF‐β after inflammatory destabilization is pivotal in promoting THO formation.^[^ ^24^ ^]^ The results of flow cytometry showed that CZCH treatment in the simulated THO microenvironment restored the M1/M2 ratio to levels similar to the Control group, compared with the IL‐1β group (Figure 2f,g). This finding may be attributed to the immunomodulatory function of curcumin in the antioxidant environment induced by CeO_2_ nanoparticles, similar to our previous findings on the M1/M2 ratio. The expression of immune‐related mRNA in macrophages treated by each group was characterized using RT‐PCR (Figure 2h). The expression of pro‐inflammatory genes TNF‐α and IL‐6 in macrophages treated with CZCH was significantly downregulated compared to the IL‐1β group and comparable to the Control group. On the other hand, the expression of IL‐10 and IL‐4 (genes associated with anti‐inflammatory responses), was significantly higher than the IL‐1β group, suggesting that CZCH may contribute to the homeostatic modulation of macrophage function in the THO microenvironment. Overall, these results indicated that CZCH induced the scavenging of excessive intracellular ROS from macrophages in the simulated THO microenvironment, reducing pro‐inflammatory gene expression and regulating the homeostasis of inflammatory‐activated macrophages. However, how CZCH achieved this function and which signaling pathways were regulated warrant further investigation.

### 2.4 Mechanism of CZCH‐Modulated Immune Homeostasis

**Figure 3:**
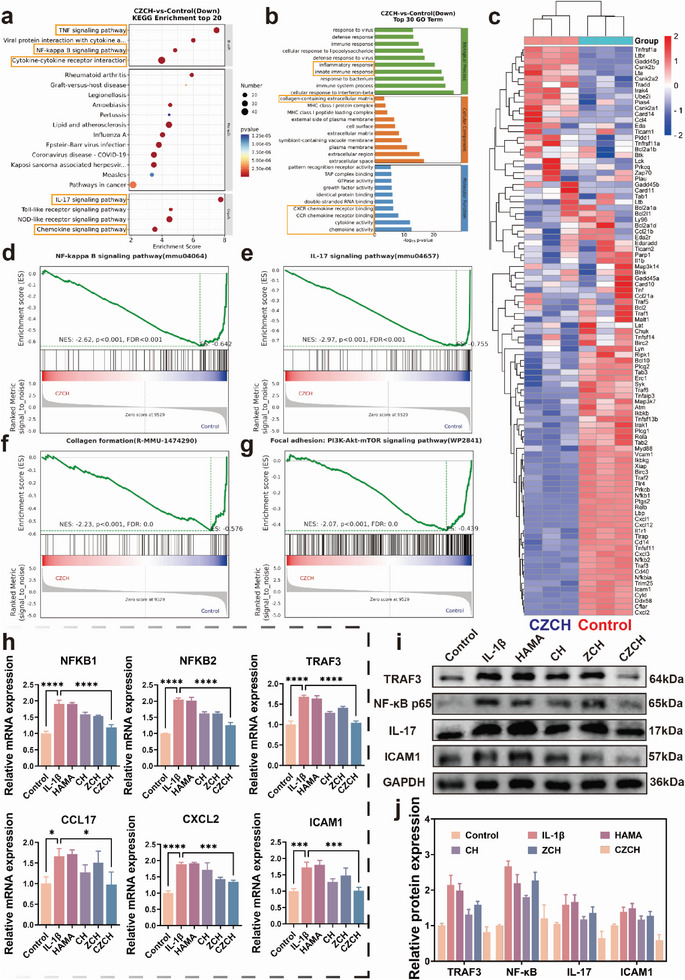
Mechanisms of CZCH modulation of immune homeostasis. a) Top 20 of downregulated differential gene KEGG enrichment pathway for CZCH compared to Control group. b) Top 30 of downregulated differential gene GO enrichment pathway for CZCH compared to Control group. c) Heat map of differential genes associated with enrichment pathway for CZCH compared to Control group. d) GSEA enrichment analysis graphs of d) NF‐κB pathway, e) IL‐17 pathway, f) collagen formation pathway, and g) PI3K‐Akt‐mTOR pathway in CZCH compared with Control group. h) Relative expression of mRNA of key signaling transcription factors of NF‐κB pathway and IL‐17 pathway in macrophages determined by RT‐PCR after co‐incubation with each group. i) WB bands of protein expression of key regulators of NF‐κB pathway and IL‐17 pathway in macrophages after co‐incubation by each group and j) corresponding relative protein quantification. *p<0.05, **p<0.01, ***p<0.001, ****p<0.0001, ns means no significance.

To further investigate the immune homeostatic mechanisms modulated by CZCH, we first applied transcriptomic mRNA sequencing (mRNA‐seq) to assess the general effect of CZCH on immune cells. Quality control results of principal component analysis (PCA), gene expression density, Fragments Per Kilobase of exon model per Million mapped fragments (FPKM) values and sample‐to‐sample distance suggested favorable group differences, demonstrating the robustness of the mRNA‐seq results (Figure S15, Supporting Information). A heatmap and volcano plot were subsequently generated, revealing that 3558 genes were upregulated and 3867 genes were downregulated after CZCH intervention, compared to the Control group (Figure S16, Supporting Information). Kyoto Encyclopedia of Genes and Genomes (KEGG) enrichment analysis and chord plot suggested that after CZCH treatment, the TNF signaling pathway, NF‐κB signaling pathway, cytokine‐cytokine receptor signaling pathway, IL‐17 signaling pathway and Chemokine signaling pathway were significantly downregulated in macrophages (**Figure** **3**a; Figure S17, Supporting Information). In contrast, GO annotation suggested that CZCH downregulated the inflammatory response, the intrinsic immune response, CXCR and CCR chemokine receptor binding function and the chemokine release function of macrophages (Figure 3b). Similar differentially expressed pathways were observed in GO chord diagram and classification (Figure S18, Supporting Information). A heatmap of the relevant differential pathways is shown in Figure 3c. GSEA enrichment analysis suggested that macrophage NF‐κB signaling pathway, IL‐17 signaling pathway, collagen formation signaling pathway and PI3K‐Akt‐mTOR signaling pathway were all downregulated by CZCH intervention (Figure 3d–g). These results suggest that the regulation of macrophage immune homeostasis by CZCH was mediated by the downregulation of the NF‐κB signaling pathway, IL‐17 signaling pathway and mTOR signaling pathway in inflammation‐activated macrophages, reducing the release of macrophage inflammatory cytokines and chemokines, thus restoring macrophage homeostasis.^[^ ^25^ ^]^ The expression of markers such as NF‐κB p65 and TRAF3 in the NF‐κB signaling pathway, IL‐17 and ICAM‐1 in the IL‐17 signaling pathway, and mTOR were verified utilizing RT‐PCR and WB. As shown in Figure 3h, the mRNA and protein expression of these representative genes were significantly downregulated after CZCH treatment compared with the IL‐1β group, validating the potential mechanism of CZCH in modulating macrophage immune homeostasis. The flow cytometry results of ICAM1 also revealed a regulatory role of CZCH (Figure S19, Supporting Information). The results of WB experiments verified the regulation of NF‐κB p65 and TRAF3 in the NF‐κB signaling pathway as well as IL‐17 and ICAM‐1 in the IL‐17 signaling pathway by CZCH. (Figure 3i,j).

### 2.5 CZCH Triggered Efferocytosis of Macrophages In Vitro

**Figure 4:**
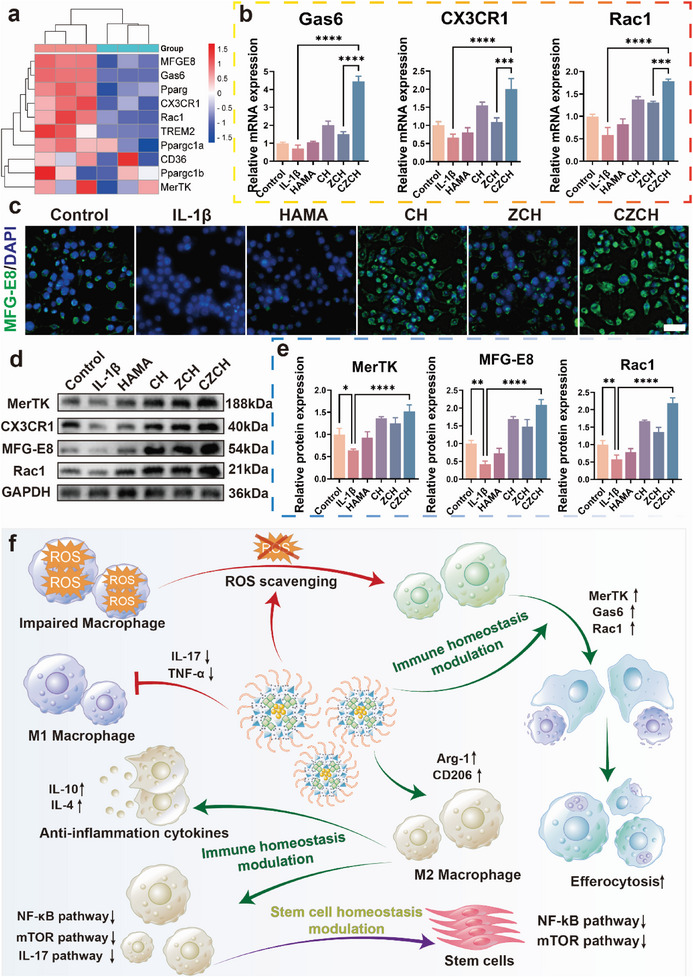
The pro‐efferocytosis effect of CZCH enhances the modulation of immune homeostasis. a) Heat map of differential genes involved in the regulation of efferocytosis effect of macrophages by CZCH and Control groups. b) Expression of mRNA related to efferocytosis effect of macrophages after co‐incubation with each group. c) Immunofluorescence image of MFG‐E8 (green) in macrophages after co‐incubation with each group. Scale bar, 50 µm. d) WB bands of efferocytosis effect proteins in macrophages after co‐incubation with each group and e) the corresponding relative protein quantification. f) Mechanistic scheme of immune homeostasis modulation by CZCH. *p<0.05, **p<0.01, ***p<0.001, ****p<0.0001, ns means no significance.

During the analysis of the mRNA‐seq results, differences in a series of efferocytosis‐related genes were observed (**Figure** **4**a). Efferocytosis is the process by which phagocytes eliminate apoptotic cells.^[^ ^26^ ^]^ Current evidence suggests that in the trauma‐induced inflammatory microenvironment, many cells undergo inflammatory apoptosis and necrosis, and these necrotic cells, if not eliminated in time, consistently induce inflammatory infiltration, thereby exacerbating the immune microenvironment dysregulation. The enhanced efferocytosis contributes to the clearance of inflammatory apoptotic cells, thus regulating immune homeostasis.^[^ ^27^ ^]^ Previous studies have demonstrated that enhanced macrophage efferocytosis remodeled the immune homeostasis of rheumatoid arthritis (RA) and reversed the RA disease process.^[^ ^28^ ^]^ Therefore, we validated representative efferocytosis genes in macrophages after CZCH intervention by RT‐PCR. As shown in Figure 4b and Figure S20 (Supporting Information), the mRNA and protein expression of the “find me” genes Mer tyrosine‐protein kinase receptor (MerTK) and CX3CR1, the “eat me” genes Gas6 and MFG‐E8, and the endocytosis gene Rac1 were significantly upregulated.^[^ ^29^ ^]^ Furthermore, IF assays showed that the expression of MFG‐E8 in macrophages was upregulated after CZCH intervention, demonstrating enhanced macrophage efferocytosis and enhanced modulation of immune homeostasis (Figure 4c). The results of WB experiments verified the upregulation of cytokine‐associated proteins MerTK, CX3CR1, MFG‐E8, and Rac1 by CZCH (Figure 4d,e).

Briefly, CZCH releases CeO_2_ nanoparticles, Zn ions and curcumin with antioxidant and immunomodulatory effects through inflammatory response degradation. Within the THO microenvironment, the scavenging of excessive intracellular ROS from inflammation‐activated macrophages, the remodeling of macrophage M1/M2 ratios, the downregulation of macrophage NF‐κB and mTOR signaling pathways, and the promotion of macrophage efferocytosis to modulate immune homeostasis (Figure 4f). However, THO is initiated by the activation of macrophages, and the subsequent triggering of stem cell homeostatic imbalance and differentiation in the wrong direction is a key factor leading to THO. Accordingly, the role of CZCH on the homeostatic modulation of stem cells in the THO microenvironment warrants further exploration.

### 2.6 CZCH‐Modulated Stem Cell Homeostasis

**Figure 5:**
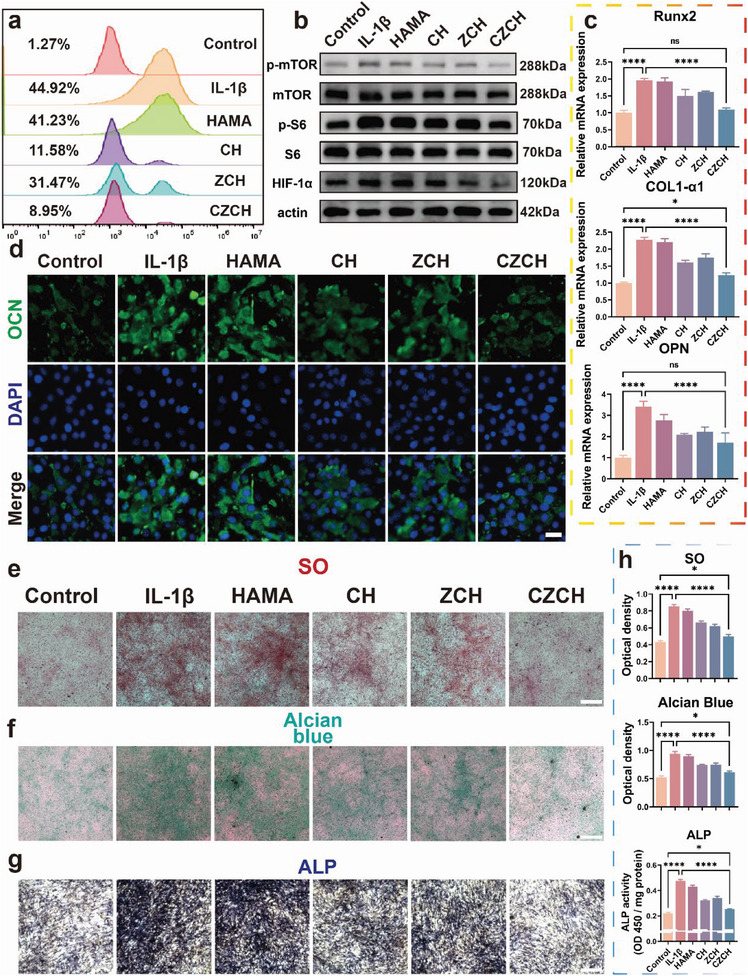
Modulation of stem cell homeostasis by CZCH in vitro. a) Flow cytometric histogram of intracellular ROS in tendon stem cells after co‐incubation with each group. b) The mTOR signaling pathway‐associated protein WB band of tendon stem cells after co‐incubation with each group. c) Relative mRNA expression of osteogenic and collagenogenic related genes in tendon stem cells after co‐incubation in each group. d) Immunofluorescence of OCN (green) in tendon stem cells after co‐incubation with each group. Scale bar, 50 µm. e) SO staining, f) Alcian blue staining, g) ALP staining, and h) their corresponding quantification of tendon stem cells after co‐incubation in each group and after osteogenesis induction. Scale bar, 250 µm. *p<0.05, **p<0.01, ***p<0.001, ****p<0.0001, ns means no significance.

To investigate the effect of CZCH on regulating stem cell homeostasis in the mimicked THO microenvironment, two types of stem cells with osteogenic differentiation potential, primary mouse tendon stem cells and mouse myoblasts stem cells C2C12, were utilized for the experiments. DCFH‐DA fluorescent probes were co‐incubated with tendon stem cells and myoblasts after each group of intervention, and the intracellular ROS content of stem cells was measured and observed using flow cytometry (**Figure** **5**a). Similar to immune cells, the intracellular ROS content of both stem cells was significantly upregulated after IL‐1β stimulation, suggesting oxidative stress. In contrast, the intracellular ROS content was attenuated compared to the Control group after CZCH intervention, indicating that the ROS scavenging ability of CZCH is applicable to immune cells and stem cells. It has been shown that CZCH could downregulate the NF‐κB and mTOR pathways in inflammation‐activated macrophages during immune homeostasis regulation. Earlier studies have established that both signaling pathways play significant roles in stem cell osteogenic differentiation, ultimately contributing to the development of THO.^[^ ^30^ ^]^ Therefore, we investigated the expression of genes of related pathways after stimulation of tendon stem cells with a conditioned medium after co‐incubation of CZCH with macrophages using WB assay. As shown in Figure 5b and Figure S21 (Supporting Information), both the NF‐κB and mTOR pathways were downregulated in tendon stem cells after intervention with an immune cell conditioned medium compared to Control and IL‐1β groups. To investigate the stem cell differentiation function after regulating stem cell homeostasis by CZCH, we further examined the osteogenic ability of tendon stem cells and myoblast. The osteogenesis‐related genes OPN, COL1‐α1, and RUNX2 in tendon stem cells were examined by RT‐PCR, and the expression of OCN in tendon stem cells was examined by IF staining. Quantification of mRNA levels of OPN, COL1‐α1, and RUNX2 also verified that CZCH inhibited the abnormal osteogenic pathological process (Figure 5c). Fluorescence images suggested that CZCH intervention reduced the expression of OCN in mouse tendon stem cells (Figure 5d). SO, Alcian blue, and ALP staining were further performed to validate the osteogenic ability of tendon stem cells. As shown in Figure 5e,h, CZCH reversed this osteogenic differentiation process compared to the osteogenic promotion induced by IL‐1β. Semi‐quantification of osteogenic staining also confirmed the antagonistic effect of CZCH on the osteogenic differentiation process of tendon stem cells.

We then examined the expression of osteogenesis‐related proteins BMP2 and RUNX2 in the myoblast cell line C2C12 by WB assay after different interventions. As shown in Figure S22 (Supporting Information), consistent with tendon stem cells, CZCH intervention downregulated the expression of osteogenic‐related proteins in C2C12 cells, suggesting regulation of stem cell homeostasis. Despite the inhibitory effect on osteoblast function, whether CZCH positively promotes the differentiation ability of stem cells needs further validation. Myogenic differentiation of C2C12 cells was observed by crystal violet staining, and the myotubular morphology of C2C12 could be observed after co‐incubation with macrophage‐conditioned medium with CZCH intervention, indicating that CZCH promotes myogenic differentiation (Figure S23, Supporting Information). The α‐SMA of C2C12 cells after incubation was examined using IF staining, and the semi‐quantitative fluorescence analysis demonstrated that CZCH promoted myofibril formation (Figure S24, Supporting Information). The macrophage‐conditioned medium after CZCH intervention also promoted the migration of fibroblasts (Figure S25, Supporting Information). In conclusion, CZCH restored stem cell homeostasis by scavenging excessive intracellular ROS, inhibiting osteogenic mis‐differentiation and promoting positive differentiation of stem cells, and facilitated the participation of stem cells in tissue reconstruction while immune homeostasis was modulated. Thus, the protective effect of CZCH against THO is mediated by the synergistic dual homeostatic modulation of immune and stem cell homeostasis.

### 2.7 Prevention of THO with CZCH in Mouse and Rat Models

**Figure 6:**
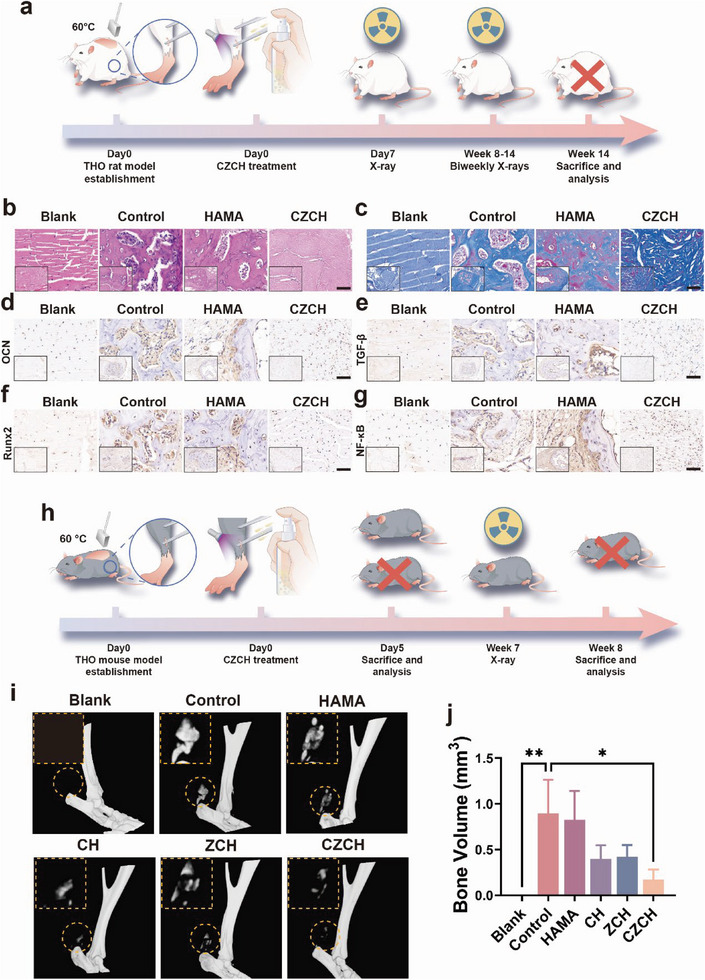
CZCH prevents tendon THO formation in rat and mouse models. a) Flow diagram of the rat THO model establishment and CZCH prevention process. b) 63x magnification of H&E stained tissue sections around the rat Achilles tendon at week 14. c) 63x magnification of Masson staining of a section of the rat Achilles tendon at week 14. d) 63x magnification of immunohistochemical staining of d) anti‐OCN, e) anti‐TGF‐β, f) anti‐Runx2, and g) anti‐NF‐κB tissue sections around the Achilles tendon of rats at week 14. The embedded images in the above figures are all 20x magnification. Scale bar, 100 µm h) Flow diagram of THO model establishment and CZCH prevention process in mice. i) Micro‐CT 3D reconstruction of the mouse Achilles tendon at week 8, and the embedded images are local THO magnification. j) Bone volume quantification of local THO in micro‐CT 3D reconstruction at the Achilles tendon in mice. *p<0.05, **p<0.01, ***p<0.001, ****p<0.0001, ns means no significance.

All animal experiments were approved by the ethics committee of our institution (2023‐N(A)−20). The flow chart of rat THO model establishment with CZCH treatment is shown in **Figure** **6**a. After the Achilles tendon THO rat model was established, it was divided into the Control group (no intervention after modeling), the HAMA group (HAMA sprayed at the trauma), the CZCH group (CZCH sprayed at the trauma), and the blank group (not modeled). The surgical procedure is shown in the Figure S26 (Supporting Information). X‐ray examination of the sections of the Achilles tendon at week 14 revealed significant THO formation in the Control group, while no significant THO formation was observed in the CZCH group (Figure S27, Supporting Information). Pathological examination of the distal tibial tissues of the rats was performed at week 14, and THO formation at the Achilles tendon, was observed in both the Control and HAMA groups during H&E and Masson staining (Figure 6b,c). In contrast, no significant THO formation was observed in the cross‐sections of the distal tibia in the CZCH group after the intervention, and there was no significant inflammatory infiltration or scar formation, suggesting a favorable tissue repair process. To assess the formation of THO, safranin O‐fast green staining was employed on cross‐sections of the rats' distal tibia (Figure S28, Supporting Information). Compared with the Control and HAMA groups, there was no significant cartilage and osteogenesis at the Achilles tendon trauma site after the CZCH group intervention, which validated the THO inhibitory ability of CZCH. Immunohistochemical (IHC) staining was performed to observe the expression of osteogenic signals in the distal tibial cross‐section. Compared with the Control and HAMA groups, the levels of osteogenic signals such as OCN, TGF‐β and RUNX2 were not upregulated at the Achilles tendon trauma site in the CZCH group, substantiating that the effects of CZCH were mediated by stem cell homeostasis (Figure 6d–f). Further, immunohistochemical staining demonstrated that the signals of NF‐κB was lower in the CZCH group and higher in the Control and HAMA groups, suggesting a potential mechanism for THO prevention by CZCH (Figure 6g).

To further validate the preventive effect of CZCH on THO, we also carried out CZCH treatment on a mouse model. The flow chart of mouse THO model establishment with CZCH treatment is shown in Figure 6h. After the mouse Achilles tendon THO model was established, it was divided into the Control group (no intervention after modeling), the HAMA group (HAMA sprayed at the trauma), the CH group (CH sprayed at the trauma), the ZCH group (ZCH sprayed at the trauma), the CZCH group (CZCH sprayed at the trauma), and the blank group (not modeled). Mice with Achilles tendon THO were assessed at week 8 using micro‐CT for the presence of THO at the Achilles tendon trauma. As shown in Figure 6i, THO at the trauma site was more pronounced in both the Control and HAMA groups compared to the blank group. The CH and CZCH groups demonstrated more significant THO inhibition, with no significant THO formation observed after CZCH intervention. Quantification of THO at the Achilles tendon using bone parametrization was further conducted (Figure 6j). Compared to the Control and HAMA groups, the CZCH group significantly downregulated bone volume (BV) at the trauma, suggesting the THO inhibition by CZCH.

### 2.8 Prevention of THO with CZCH via DHM Strategy In Vivo

**Figure 7:**
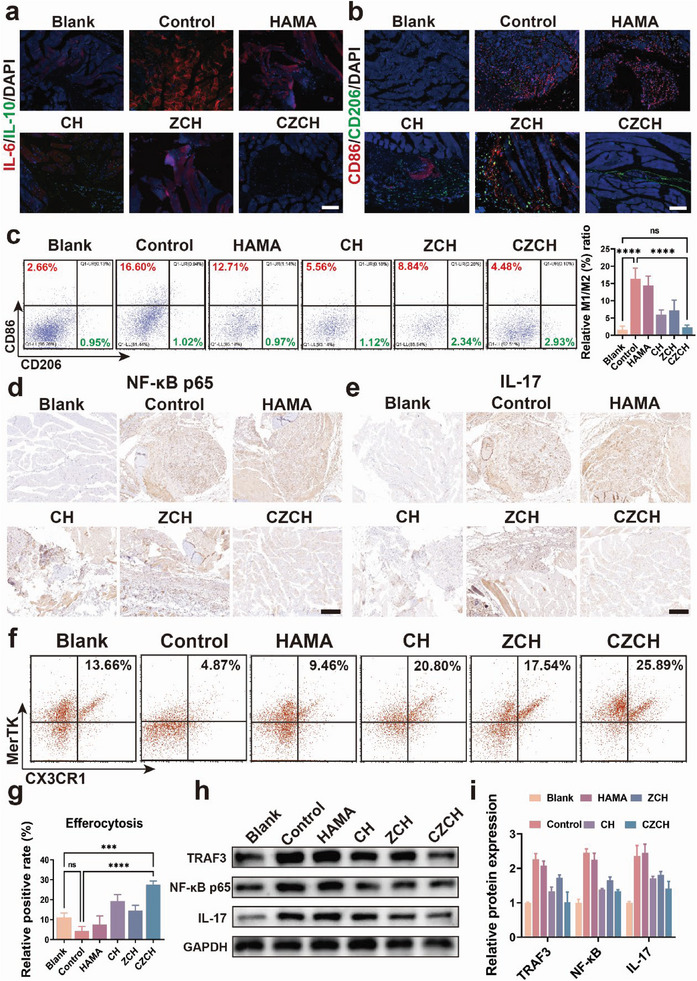
Prevention of tendon THO formation by CZCH through DHM strategy in vivo. a) Immunofluorescence images of anti‐IL‐6 (red) and anti‐IL‐10 (green) in tissue sections around the Achilles tendon of each group of mice on day 5 after modeling. Scale bar, 200 µm. b) Immunofluorescence of anti‐CD86 (red) and anti‐CD206 (green) in tissue sections around the Achilles tendon of each group of mice on day 5 after modeling. Scale bar, 200 µm. c) Flow cytograms of CD206 (M2) and CD86 (M1) in single‐cell suspensions of tissue around the Achilles tendon of each group on day 5 after modeling and the corresponding relative positivity ratios (%). d) Immunohistochemical plots of anti‐NF‐κB p65 and e) anti‐IL‐17 in tissue sections around the Achilles tendon of each group on day 5 after modeling. Scale bar, 500 µm. f) Flow cytograms of MerTK and CX3CR1 in single‐cell suspensions of peri‐Achilles tendon tissues from each group on day 5 after modeling and g) the corresponding percentage of double‐positive cells (%). h) WB bands of NF‐κB p65 and IL‐17 signaling pathway‐related proteins in homogenates of tissue around the Achilles tendon of each group of mice on day 5 after modeling and i) the corresponding relative protein expression quantification. *p<0.05, **p<0.01, ***p<0.001, ****p<0.0001, ns means no significance.

To clarify the specific mechanism by which CZCH inhibits THO formation in vivo, we investigated it based on the mouse THO model. The distal tibia of mice on day 5 after CZCH intervention was harvested and underwent histopathological analysis. As shown in Figure S29 (Supporting Information), the inflammatory infiltrate was significantly decreased in the cross‐sectional H&E staining of the distal tibia of mice after CZCH intervention. Compared with the Control and HAMA groups, the expression of cytokines such as IL‐6 was significantly downregulated after CZCH intervention (**Figure** **7**a). The results of IF were also verified by ELISA to detect cytokines in the surrounding tissue homogenates (Figure S30, Supporting Information). Meanwhile, the M1/M2 ratio was similar to the blank group after CZCH intervention, and the expression of M1 marker CD86 was significantly downregulated compared to the Control and HAMA groups (Figure 7b). To further validate the regulatory mechanism of CZCH, we examined the cellular markers within the soft tissue cell suspension around the distal tibia of mice on day 5 using flow cytometry. The M1/M2 ratio of macrophages was similar to the results of immunofluorescence sections, suggesting the modulation of immune homeostasis by CZCH (Figure 7c; Figure S31, Supporting Information). To investigate the CZCH‐induced alterations in signaling pathways, we utilized IHC staining for the key transcription factors validated by RNA‐seq and WB in the in vitro study. As shown in Figure 7d,e and Figures S32 and S33 (Supporting Information), the expression of NF‐κB, IL‐17, ICAM1, and mTOR were significantly downregulated in the distal tibial cross‐section of mice after CZCH intervention, demonstrating the strong inhibitory ability of CZCH on trauma‐induced inflammatory activation in the early stages of THO. Meanwhile, the expression of both macrophage efferocytosis markers MerTK and CX3CR1 were significantly upregulated after CZCH intervention via flow cytometry, suggesting CZCH could promote macrophage efferocytosis action by modulating immune homeostasis (Figure 7f,g). Lastly, animal proteins were extracted from tissue homogenates at the Achilles tendon of mice for WB assays to verify the regulation of NF‐κB signaling pathway by CZCH (Figure 7h,i). Taken together, these findings corroborated that CZCH prevented THO formation via the DHM approach in vivo.

Finally, the biosafety of CZCH was evaluated for potential in vivo application. No significant abnormalities were found in the blood (erythrocyte count, white blood cell count, neutrophil count and lymphocyte count, Figure S34, Supporting Information) and liver and kidney functions (glutamic oxalacetic transaminase (AST), glutamic alanine transaminase (ALT), urea nitrogen and creatinine, Figure S35, Supporting Information) of the mice after treatment in each group. The in vivo biocompatibility of CZCH was evaluated by H&E staining of the heart, liver, lung, spleen, and kidney in each group of mice at week 8 and at week 15 post‐intervention. No pathological changes or adverse effects were detected in mice and rats treated with CZCH, further validating the high biosafety profile of CZCH (Figure S36, Supporting Information). Combined with in vivo biosafety assessment and favorable THO prophylaxis, CZCH develops no additional damage to peripheral tissues and vital organs, providing a satisfactory long‐term biosafety.

## 3 Conclusion

Our current study suggests a DHM strategy that harnesses the pathophysiological characteristics of dysregulated microenvironmental homeostasis and abnormal inflammatory activation of THO. Inhibition and synergistic prevention of THO during the early stages of trauma were achieved by inducing stem cell homeostasis via the modulation of immune homeostasis. We designed a CZCH hydrogel based on DHM strategy that could be extensively sprayed and rapidly photocured in situ, convenient for cases of trauma areas which could form hydrogel films to induce the inflammatory response. It was verified that CZCH inhibited THO formation by promoting macrophage repolarization and efferocytosis through NF‐κB, mTOR, IL‐17, and other signaling pathways as well as inducing normal differentiation of stem cells. Our findings substantiate that CZCH has huge prospects for application in clinical practice and pioneered the hydrogel spray application for THO prevention.

## 4 Experimental Section

### Synthesis and Characterization of CZC

CZC was synthesized according to a previously reported procedure with some modifications. Specifically, 500 mg of 2‐methylimidazole and 5 µg of curcumin were mixed in 4 mL of methanol and stirred for 10 min at room temperature. Then 1 mL of a water solution containing 2.5 mg Zn(NO_3_)_2_.6H_2_O was added to the above mixture. The reaction was stirred vigorously for 15 min to obtain an emulsion‐like suspension. The nano‐cerium oxide particles and polyvinylpyrrolidone were dispersed in methanol and added to the above emulsion‐like suspension, and the reaction is stirred for 5 min at room temperature. The final product of the reaction was obtained after centrifugation and impurities are removed by washing with methanol. ZC was synthesized using the same procedure. For characterization, the morphology and particle size of CZC NPs were observed using TEM (F Tecnai F20 microscope; Holland); the zeta potential and particle size distribution of CZC NPs were measured using a zeta potential and nanoparticle size analyzer (Nano ZS90, Malvern; UK); the distribution and proportion of elements Zn and Ce in CZC NPs were detected using EDS (JEOL JEM 2100F; Japan); the chemical relationships of the components in CZC NPs were examined using FT‐IR (Nicolet iS 10; US); the crystal conformation of CZC was analyzed using XRD (X' Pert PRO MPD; Holland); XPS (Axis Ultra DLD Kratos AXIS SUPRA; UK) was used to examine the valence states of the elements Zn and Ce in CZC NPs.

### Synthesis and Characterization of CZCH

HAMA was purchased from Engineer for Life company (China). To synthesize CZCH, 1 mg of HAMA was initially dissolved in the photoinitiator lithium phenyl‐2,4,6‐trimethylbenzoylphosphinate (LAP) (10 mL; 0.25%; w/v) and stirred at room temperature for 20 min. Subsequently, a certain proportion of curcumin, ZC, and CZC solutions was added to the HAMA gels, vortexed for 10 min, and then sonicated for 10 min. Finally, each hydrogel system was added to a spray bottle, the cap was pressed to make a mist spray, and the hydrogel spray was irradiated with a 405 nm light source to solidify it. For characterization, the morphological characteristics and elemental distribution of CZCH were observed using an SEM (ZEISS GeminiSEM 300; Germany); the successful synthesis of CZCH was verified using FT‐IR; the rheological properties of the hydrogels were determined using a rheological analyzer (Discovery HR‐2; USA). To assess the swelling properties of the hydrogels, 1 mL of hydrogel with an initial mass of W_0_ was spray‐cured and then immersed in PBS. The hydrogel was taken out at the predefined time point, and its mass was recorded as W_t_ after wiping off the surface PBS. The swelling rate of the hydrogel was calculated according to Equation (1):

(1) $$\mathrm{Swelling}\;\mathrm{ratio}=\left(W_{t}-W_{0}\right)/W_{0}\times 100\%$$

To assess the degradation characteristics of the hydrogels under different conditions, 1 mL of hydrogel with an initial mass noted as W_0_ was spray‐cured. It was immersed in PBS until fully swollen and then transferred to three different solution systems: neutral PBS (pH = 7.4), acidic PBS (pH = 5), and PBS supplemented with 10% fetal bovine serum (FBS) (pH = 7.4). The degraded residual hydrogel was taken out and blotted dry at the set time points, and its mass was recorded as Wp. The degradation rate of the hydrogel was calculated according to Equation (2):

(2) $$\mathrm{Degradation}\;\mathrm{ratio}=\left(W_{0}-W_{p}\right)/W_{0}\times 100\%$$

### Zn and Curcumin Release Assay

CZCH hydrogel(1 mL) was spray‐cured and immersed in neutral PBS solution (pH = 7.4), acidic PBS solution (pH = 5), neutral H_2_O_2_ solution (1 m; pH = 7.4), and acidic H_2_O_2_ solution (1 m; pH = 5), respectively. The supernatant was collected and centrifuged at set time points, and the concentrations of Zn and curcumin within it were determined using ICP‐MS (PerkinElmer Optima 5300 DV ICP‐OES; USA) and UV–vis (PerkinElmer Lambda 750; USA), respectively.

### ROS Scavenging Assay

Tetramethylbenzidine (TMB; Aladdin; China) was employed to evaluate the ROS‐scavenging effect of CZCH. Briefly, 1 mL of CZCH hydrogel was spray‐cured and immersed in PBS until completely degraded. The hydrogel degradation solution was mixed 1:1 with TMB solution (1 mm TMB in an acetate buffer; pH = 5), followed by hydrogen peroxide solution (1 m). At set time points, the absorbance of the reaction solution was measured in the 500 nm to 800 nm interval using a UV–vis spectrophotometer (PerkinElmer Lambda 750; USA).

### Cell Culture

In this study, mouse macrophage cell lines (RAW 264.7), mouse tendon stem cells (CP‐M176), mouse myogenic cells (C2C12), mouse fibroblasts (L929), human umbilical vein cell fusion cells (Ea. hy926), and mouse 3T3 osteoblasts were purchased from Pricella (China). All cells were cultured in a constant temperature incubator at 37 °C with 5% CO_2_. The cell culture status was observed daily, and the medium was changed every 1–2 days. Passaging was performed when the cell density in the culture dish reached 80–90%.

### Preparation of Hydrogel‐Conditioned Medium and Macrophage‐Conditioned Medium

The hydrogel‐conditioned medium (HCM) was used to culture macrophages. Specifically, the hydrogels were spray‐cured and immersed in a DMEM medium (Gibco; US) at various volume ratios. After complete hydrogel degradation, 10% FBS (Gibco; US) and 1% penicillin/streptomycin (Gibco; US) were added to the above mix. The HCM medium was then filtered using a 0.22 µm filter (Merck; Germany) and frozen at −20 °C for storage. The HCM was collected after culturing macrophages and named macrophage conditioned medium (MCM). Specifically, RAW264.7 cells were inoculated into 6‐well plates at a density of 1 × 10^6^ per well, and the cells were divided into six groups according to the treatment conditions: control, IL‐1β, IL‐1β + HAMA, IL‐1β + CH, IL‐1β + ZCH, and IL‐1β + CZCH groups. After cell apposition, the medium was discarded, and the cells were gently washed with PBS. The control and IL‐1β groups were cultured with DMEM complete medium with or without IL‐1β (10 ng mL^−1^; Thermo; US), while the other groups were cultured with the corresponding HCM containing IL‐1β (10 ng mL^−1^). The culture medium from each group was collected after 24 h, filtered with a 0.22 µm filter, and mixed with DMEM complete medium at a volume ratio of 1:2 for other cell cultures.

### Biocompatibility Assay

To test the cytotoxicity of CZCH, a hydrogel‐conditioned medium containing different concentrations of CZC was first prepared, as described previously. RAW264.7 cells were inoculated into 96‐well plates at a density of 1 × 10^5^ per well. After cell apposition, the original medium was replaced with HCM, and the cells were cultured for 24 h. Afterward, cells were gently washed with PBS and incubated in DMEM basal medium containing 10% CCK‐8 reagent (Beyotime; China) for 30 min. Finally, the absorbance at 450 nm was measured using a microplate reader (BIO‐TEK; USA), and the relative viability of the cells was calculated according to Equation (3):

(3) $$\mathrm{Cell}\;\mathrm{viability}=\left(\mathrm{ODs}-\mathrm{ODc}\right)/\mathrm{ODc}\times 100\%$$

where *ODs* and *ODc* are the optical density (OD) values of the sample and control, respectively.

To test the hemocompatibility of CZCH, fresh whole blood was collected from healthy mice. Red blood cells (RBC) were obtained by centrifugation and further resuspended using PBS. Hydrogel degradation solutions containing different concentrations of CZC were mixed with RBC dispersions and incubated at 37 °C for 1 h. PBS and ddH_2_O‐treated groups were used as negative and positive controls, respectively. All samples were centrifuged, and the supernatant was transferred to a 96‐well plate. The absorbance at 540 nm was measured using a microplate reader (BIO‐TEK; USA), and the relative hemolysis rate was calculated according to Equation (4):

(4) $$\mathrm{Hemolysis}\;\mathrm{ratio}=\left[\left(\mathrm{Abs}0-\mathrm{Absn}\right)/\left(\mathrm{Absp}-\mathrm{Absn}\right)\right]\times 100\%$$

where *Abs0*, *Absn*, and *Absp* denote the absorbance of the sample, negative control, and positive control, respectively.

### Cellular ROS Assay

After various treatments, the cells were washed with PBS, and the ROS fluorescent probe DCFH‐DA (1:1000 dilution; Beyotime; China) was added. After incubation for 30 min at 37 °C in an incubator protected from light, a DM8 fluorescence microscopy (Leica; Germany) was used to observe the cells, or a flow cytometer (Beckman Coulter; USA) was used to determine the fluorescence intensity of ROS in the cells.

### Flow Cytometry In Vitro

The treated cells were washed with PBS and incubated with fluorescein‐labeled flow antibodies on ice, protected from light. 30 min later, the cells were washed and resuspended using PBS, and the fluorescence intensity of the targeted marker was measured using flow cytometry. Flowjo software was finally used to analyze the data. The flow cytometry antibodies used in vitro for this study are listed in Table S1 (Supporting Information).

### Immunofluorescence Staining In Vitro

After discarding the culture medium, the treated cells were washed with PBS, fixed with 4% paraformaldehyde, and blocked with an immunostaining blocking solution (Beyotime; China). Afterward, primary antibodies to the target molecules were added to the cells and incubated at 4 °C overnight. The following primary antibodies were used for the immunofluorescence staining in vitro: OCN, MFGE8, CCR7, and Arg‐1. All the primary antibodies were purchased from the ABclonal company in China. The cells were washed with PBS and incubated with fluorescently labeled secondary antibodies of the corresponding species at room temperature for 1 h. The nuclei were stained with 2‐(4‐Amidinophenyl)−6‐indolecarbamidine dihydrochloride (DAPI) (Beyotime; China). Finally, the cells were observed using a DM8 fluorescent microscope, and the relative fluorescence intensity was calculated using ImageJ software.

### RT‐PCR Assay

The culture medium was discarded, and the treated cells were washed with PBS. Total cellular RNA was extracted using the RNA extraction kit (EZBioscience; USA) according to the manufacturer's instructions. The cDNA was synthesized using the reverse transcription kit (EZBioscience; USA) according to the corresponding RNA concentration determined by Nanodrop 2000 (Thermo; US). 10 ul of the reaction system was finally prepared for PCR detection using the PCR kit (EZBioscience; USA). The Ct values were read, and GAPDH was used as a reference to analyze gene expression levels. The primers for the genes used in this study are shown in Table S2 (Supporting Information).

### ELISA Assay

Cell culture medium or tissue homogenate was collected and centrifuged to obtain the supernatant. According to the manufacturer's instructions, the ELISA assay kit (Dakewe Biotech; China) was used to detect the secretion of inflammation‐related cytokines, including TNF‐α, IL‐6, IL‐4, and IL‐10.

### Transcriptomic Analysis

RAW264.7 cells were inoculated at a density of 1 × 10^6^ per well into 6‐well plates. When the cells were grown to 80% confluence, the original medium was discarded, and a fresh hydrogel medium was added according to the grouping. After 24 h of incubation, the total cellular RNA was extracted using Trizol (Beyotime; China) and immediately frozen in liquid nitrogen. The libraries were sequenced on a Ilumina Novaseq 6000 platform and 150 bp paired‐end reads were generated. Raw reads of fastq format were first processed using fastp and the low‐quality reads were removed to obtain the clean reads. The clean reads were mapped to the reference genome using HISAT2. FPKM of each gene was calculated and the read counts of each gene were obtained by HTSeq‐count. PCA analysis were performed using R (v 3.2.0) to evaluate the biological duplication of samples. Differential expression analysis was performed using the DESeq25. Q value < 0.05 and foldchange > 2 or foldchange < 0.5 was set as the threshold for significantly differential expression gene (DEGs). Hierarchical cluster analysis of DEGs was performed using R (v 3.2.0) to demonstrate the expression pattern of genes in different groups and samples. The radar map of top 30 genes was drawn to show the expression of up‐regulated or down‐regulated DEGs using R packet ggradar. Based on the hypergeometric distribution, GO, KEGG pathway, Reactome and WikiPathways enrichment analysis of DEGs were performed to screen the significant enriched term using R (v 3.2.0), respectively. R (v 3.2.0) was used to draw the column diagram, the chord diagram and bubble diagram of the significant enrichment term. Gene Set Enrichment Analysis (GSEA) was performed using GSEA software.

### Western Blot (WB) Assay

The treated cells were washed with PBS, and the RIPA cell lysate (EpiZyme; China) was added, shaking at 4 °C for 30 min. The cell lysate was collected into EP tubes and centrifuged, and the protein concentration in the supernatant was determined using a BCA kit. The extracted proteins were separated using sodium dodecyl sulfate‐polyacrylamide gel electrophoresis (SDS‐PAGE) (EpiZyme; China) and transferred to polyvinylidene difluoride (PVDF) membranes (Beyotime; China). The membranes were blocked in a blocking solution (Beyotime; China) for 15 min, washed in TBST (Servicebio; China), and incubated with the primary antibody overnight at 4 °C. After washing again, membranes were incubated with secondary antibodies for 1 h at room temperature. Blots were detected by chemiluminescence using enhanced chemiluminescence reagents (Thermo; USA) and the Tanon imaging system (Tanon; China). Relative band intensities were quantified using ImageJ software, and all results were normalized to GAPDH or Actin expression. The WB antibodies used in this study are shown in Table S3 (Supporting Information).

### Myogenic Assay

C2C12 cells were inoculated at a density of 2 × 10^5^ per well into 24‐well plates. When the cells were grown to 80% confluence, the original medium was discarded, and a macrophage‐conditioned medium (containing 2% horse serum) was added for differentiation induction. The medium was changed daily, and cell morphology and growth were observed under the microscope. Induction was completed when thick tubular structures appeared.

### Scratching Assay

L929 cells were inoculated into 24‐well plates at a density of 2 × 10^5^ per well. When the cells were fully grown, a 20 µL pipette was used to create cell scratches perpendicular to the well plate. Microscopic photographs are taken after the scratch is complete and serve as a 0 h control. The original medium was aspirated and a macrophage‐conditioning medium was added. After 24 h of incubation, the width of the scratch was observed under the microscope and photographed. The scratch widths at 0 h and 24 h were measured using ImageJ software, and cell migration rates were calculated.

### Osteogenic Staining

The 3T3 cells were inoculated into 48‐well plates at a density of 1 × 10^5^ per well. When the cells have grown to 80% confluence, the original medium is discarded, and the osteogenic medium is added to induce osteogenic differentiation. The osteogenic induction medium consisted of the macrophage‐conditioned medium, 10^−8^ m dexamethasone (Beyotime; China), vitamin C (50 µg mL^−1^) (Beyotime; China), and 10 mm β‐phosphoglycerol (Beyotime; China). After 7 days of culture, the cells were stained and quantified for ALP using the ALP assay kit (Beyotime; China).

### Chondrogenic Staining

CP‐M176 cells were inoculated at a density of 1 × 10^5^ per well into 48‐well plates. When the cells grew to 80% confluence, the original medium was discarded, and the chondrogenic medium was added to induce chondrogenic differentiation. The chondrogenic induction medium consisted of the macrophage‐conditioned medium, 1 mm sodium pyruvate, vitamin C (50 µg mL^−1^), 10^−7^ m dexamethasone, 1% Insulin‐Transferrin‐SelenIum (Beyotime; China), and TGF‐β3 (10 ng mL^−1^) (Merck; Germany). After 14 days of culture, cells were stained using Alcian blue (Beyotime; China) and Oil Red O (Servicebio; China). Micrographs were analyzed for optical density using ImageJ.

### Burn/Tenotomy HO Model

All animal experiments were approved by the ethics committee of our institution (2023‐N(A)−20). The burn/tenotomy HO model was established by the same orthopedic surgeon using ICR mice (male, 8–10 weeks old) and SD rats (male, 8–10 weeks old) according to a previously described protocol. Briefly, the animals were anesthetized with 1% pentobarbital intraperitoneally, and the dorsal hair was shaved. Afterward, a block of the aluminum heated to ≈60 °C was exposed for 17 s to an area covering more than 30% of the total body surface of the dorsal area to create a partial thickness burn. All animals then underwent aseptic hindlimb tendon transection at the midpoint of the Achilles tendon, and CZCH hydrogel were sprayed on the wound and photo‐crosslinked with a 405 nm light source, and the skin was closed with sutures. The unoperated group was used as a blank and the operated untreated group as a control. Pain management was achieved by subcutaneous injection of buprenorphine every 12 h for 2 days. For mice, some of the lower legs were harvested for histological analysis 5 days after surgery, and the remaining were subjected to Micro‐CT analysis 8 weeks after surgery. All rats were euthanized 14 weeks after surgery, and the lower legs were taken for X‐ray scanning and subsequent histological analysis.

### Micro‐CT Analyses

The mouse hind limbs were fixed in 4% paraformaldehyde and analyzed with Micro‐CT (Skyscan1172; Belgium) at a voltage of 80 kV and 18 µm per pixel resolution. Images were reconstructed, visualized, and analyzed for bone volume by NRecon, CTAn, and CTVol software.

### X‐Ray Scans

A digital X‐ray machine (PLX7100A; China) was used to take front and side views of each specimen with an exposure time of 6000 ms and a voltage of 32 kV.

### Histology, Immunohistochemistry, and Immunofluorescence In Vivo

The hind limbs of the animals were fixed in 4% paraformaldehyde and sequentially dehydrated in 50%, 70%, 80%, 90%, 95%, and 100% ethanol solutions for 20 min each time. The tissue was embedded in paraffin after 1 h of transparency using xylene. The wax blocks were trimmed, fixed on a paraffin slicer, and sectioned at 5 µm thickness. The sections were unfolded in water and finally attached to slides for drying. For HE, Masson, and Safranin O‐Fast Green staining, paraffin sections were dewaxed in xylene, hydrated with gradient ethanol, and then stained using standard HE, Masson, and Safranin O‐Fast Green staining procedures. For IHC and IF assays, sections were incubated with primary antibodies overnight at 4 °C. The following primary antibodies were used in this study: IL‐6, IL‐10, CD86, CD206, NF‐κB p65, IL‐17, OCN, TGF‐β, and RUNX2. All the primary antibodies were purchased from the ABclonal company in China. For IHC, sections were incubated with horseradish peroxidase‐conjugated secondary antibodies for 1.5 h, stained with 3,3′diaminobenzidine solution. For IF, sections were subsequently incubated with fluorescein‐conjugated secondary antibodies for 1.5 h and observed under a fluorescent microscope.

### Flow Cytometry In Vivo

Achilles tendons and surrounding tissues were harvested immediately after euthanasia. The tissue was mechanically cut into small pieces and further digested with 1 mg mL^−1^ collagenase IV (Gibco; US), 1 mg mL^−1^ collagenase I (Gibco; US), and 100 µg mL^−1^ deoxyribonuclease (Gibco; US) at 37 °C for 1 h. After filtration through a 70 µm filter, single cells were collected and treated with ACK lysis buffer (Gibco; US) for 5 min at room temperature to lyse the erythrocytes. The single cell suspension was incubated with an anti‐CD16 /32 antibody for 15 min to block the Fc receptor and further incubated with a fluorescein‐labeled antibody for 45 min on ice. Finally, the fluorescence intensity was detected using flow cytometry, and the data were analyzed using FlowJo software. The flow cytometry antibodies used in vivo for this study are listed in Table S1 (Supporting Information).

### Statistical Analysis

All data in this study are analyzed using GraphPad Prism 9.2 software and presented as the means ± standard (mean ± SD) deviation. One‐way ANOVA and Student's *t* test were employed to determine the statistical significance, which was set at *p* < 0.05. Study‐specific analyses are reported in figure captions.

## Conflict of Interest

The authors declare no conflict of interest.

## Supporting information

Supporting Information

## Data Availability

The data that support the findings of this study are available from the corresponding authors upon reasonable request.
