# Supplementary material for: Inflammation‐Responsive Hydrogel Spray for Synergistic Prevention of Traumatic Heterotopic Ossification via Dual‐Homeostatic Modulation Strategy
Source: Adv Sci (Weinh). 2023 Aug 27;10(30):2302905. doi: 10.1002/advs.202302905 (PMC10602522; doi:10.1002/advs.202302905)
Supplement: Supplementary file 1 — Supporting Information [file ADVS-10-2302905-s001.pdf]

ADVANCED SCIENCE
Open Access

Supporting Information

for *Adv. Sci.*, DOI 10.1002/advs.202302905

Inflammation-Responsive Hydrogel Spray for Synergistic Prevention of Traumatic Heterotopic Ossification via Dual-Homeostatic Modulation Strategy

Jiazhao Yang, Xudong Zhang, Baoliang Lu, Jiawei Mei, Lei Xu, Xianzuo Zhang, Zheng Su, Wei Xu, Shiyuan Fang, Chen Zhu*, Dongdong Xu* and Wanbo Zhu**

Supplement Information

Inflammation-responsive hydrogel spray for synergistic prevention of traumatic heterotopic ossification via dual-homeostatic modulation strategy

Jiazhao Yang[1#], Xudong Zhang[1#], Baoliang Lu[1#], Jiawei Mei[1], Lei Xu[1], Xianzuo Zhang[1], Zheng Su[1], Wei Xu[1], Shiyuan Fang[1], Chen Zhu[1*], Dongdong Xu[2*] and Wanbo Zhu[2*]*

1: J. Yang, XD. Zhang, B. Lu, J. Mei, L. Xu, XZ. Zhang, Z. Su, W. Xu, S. Fang, C. Zhu
Department of Orthopedics
The First Affiliated Hospital of USTC
University of Science and Technology of China, Hefei,
Anhui 230001, P. R. China
E-mail: fangshiyuan@ustc.edu.cn; zhuchena@ustc.edu.cn

2: D. Xu, W. Zhu
Department of Orthopedics
Shanghai Jiao Tong University Affiliated Sixth People's Hospital
Shanghai Jiao Tong University
Shanghai 200233, P. R. China
E-mail: xdd0529@sjtu.edu.cn; zhuwanbo@sjtu.edu.cn

#These authors contributed equally to this work.

1. Supplementary tables

Supplementary table 1. Flow cytometry antibodies used in vitro and in vivo for this study

	Color	Antibody	Company	Cat#	Country
In vitro	APC	CCR7	BioLegend	120107	China
	PE	CD206	BioLegend	141705	China
In vivo	APC	CD86	BioLegend	105113	China
	PE	CD206	BioLegend	141705	China
	APC	MerTK	BioLegend	151507	China
	FITC	CX3CR1	BioLegend	149020	China

Supplementary table 2. Gene primers used in this study

	Primer	Sequence (5′−3′)
Cell	RUNX2-F	ATGCTTCATTCGCCTCACAAA
	RUNX2-R	ATGCTTCATTCGCCTCACAAA
	COL1-α1-F	GCTCCTCTTAGGGGCCACT
	COL1-α1-R	CCACGTCTCACCATTGGGG
	OPN-F	AGCAAGAAACTCTTCCAAGCAA
	OPN-R	GTGAGATTCGTCAGATTCATCCG
	Gas6-F	TGCTGGCTTCCGAGTCTTC
	Gas6-R	CGGGGTCGTTCTCGAACAC
	CX3CR1-F	ACGAAATGCGAAATCATGTGC
	CX3CR1-R	CTGTGTCGTCTCCAGGACAA
	Rac1-F	GAGACGGAGCTGTTGGTAAAA
	Rac1-R	ATAGGCCCAGATTCACTGGTT
	NF-κB1-F	ATGGCAGACGATGATCCCTAC
	NF-κB1-R	TGTTGACAGTGGTATTTCTGGTG
	NF-kB2-F	GGCCGGAAGACCTATCCTACT
	NF-kB2-R	CTACAGACACAGCGCACACT
	TRAF3-F	GTAGGACTGGAGTACGTGTGG
	TRAF3-R	TCTTCCACCGTCTTCACAAAC
	CCL17-F	TACCATGAGGTCACTTCAGATGC
	CCL17-R	GCACTCTCGGCCTACATTGG

	CXCL2-F	CCAACCACCAGGCTACAGG
	CXCL2-R	GCGTCACACTCAAGCTCTG
	ICAM1-F	GTGATGCTCAGGTATCCATCCA
	ICAM1-R	CACAGTTCTCAAAGCACAGCG
	TNF-α-F	ACTGAACTTCGGGGTGATCG
	TNF-α-R	TGGTGGTTTGTGAGTGTGAGG
	IL-6-F	TTCCATCCAGTTGCCTTCTTG
	IL-6-R	TCATTTCCACGATTTCCCAGAG
	GAPDH-F	AGGTCGGTGTGAACGGATTTG
	GAPDH-R	TGTAGACCATGTAGTTGAGGTCA
	RNAIII-F	GAATTTGTTCACTGTGTCGATAATCCATTT
	RNAIII-R	GAAGGAGTGATTTCAATGGCACAAGATAT
	sarA-F	GCTGTATTGACATACATCAGCGAAA
	sarA-R	CGTTGTTTGCTTCAGTGATTCGT

Supplementary table 3. WB antibodies used in this study

	Antibody	Company	Cat#	Country
Primary antibodies	mTOR Rabbit pAb	Abclonal	A2445	China
	p-mTOR Rabbit pAb	Abclonal	AP0094	China
	S6 Rabbit pAb	ZENBIO	R25622	China
	p-S6 Rabbit pAb	ZENBIO	310090	China
	HIF-1α Rabbit pAb	Abclonal	A22041	China
	MerTK Rabbit pAb	Abclonal	A12294	China
	CX3CR1 Rabbit pAb	Abclonal	A2890	China
	MFGE8 Rabbit pAb	Abclonal	A12322	China
	Rac1 Rabbit pAb	Abclonal	A5080	China
	TRAF3 Rabbit pAb	Abclonal	A15106	China
	NF-κB p65 Rabbit pAb	Abclonal	A2547	China
	IL-17 Rabbit pAb	Abclonal	A0688	China
	ICAM1 Rabbit pAb	Abclonal	A5597	China
	BMP2 Rabbit pAb	Abclonal	A14708	China
	RUNX2 Rabbit pAb	Abclonal	A11753	China
	GAPDH Rabbit pAb	Abclonal	AC001	China
	β-Actin Rabbit pAb	Abclonal	AC006	China
Second antibody	HRP Goat Anti-Rabbit IgG (H+L)	Abclonal	AS014	China

2. Supplementary figures

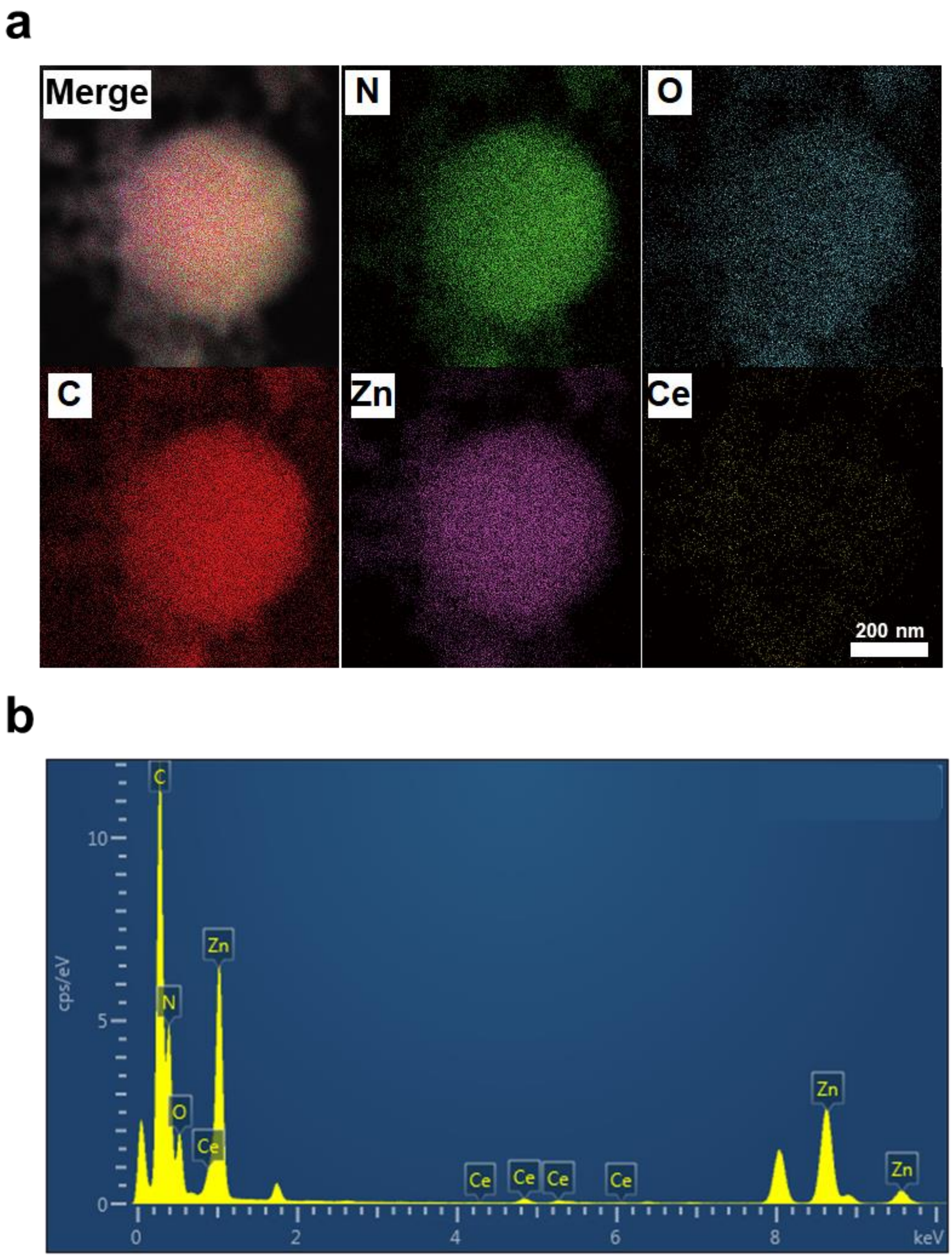

Figure S1. Elemental characterization of Cur@ZIF@CeO_2 (CZC). (a) Transmission electron microscope image and energy spectrum mapping of CZC and (b) EDS of CZC. Scale bar, 200 nm.

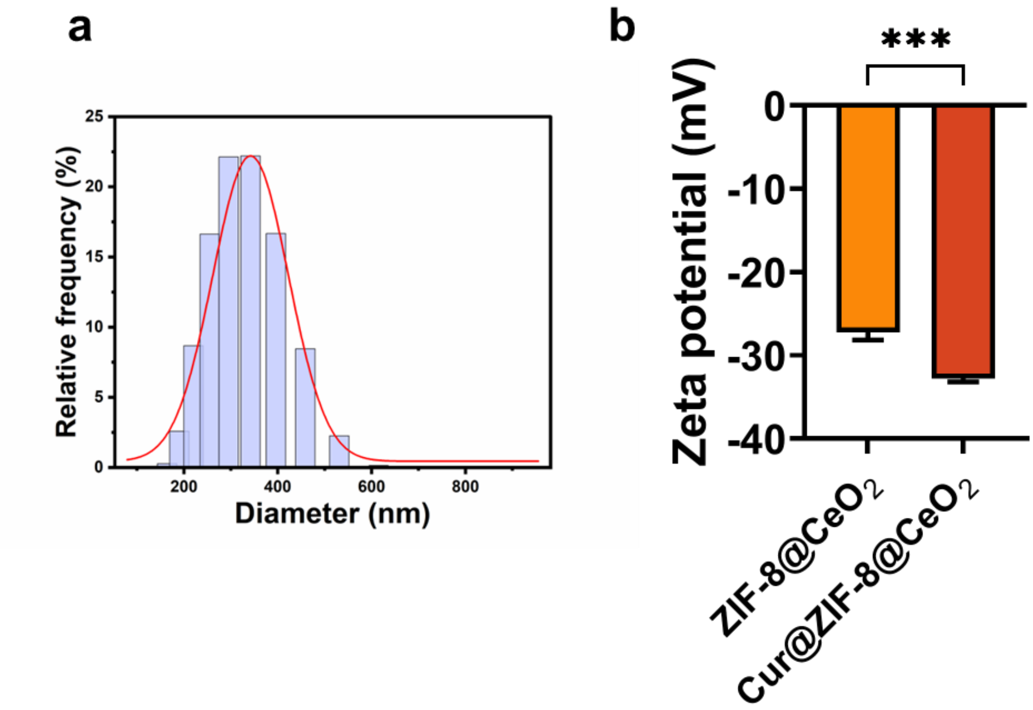

Figure S2. (a) DLS of CZC and (b) zeta potential of ZIF-8@CeO_2 and CZC. ****p*<0.005.

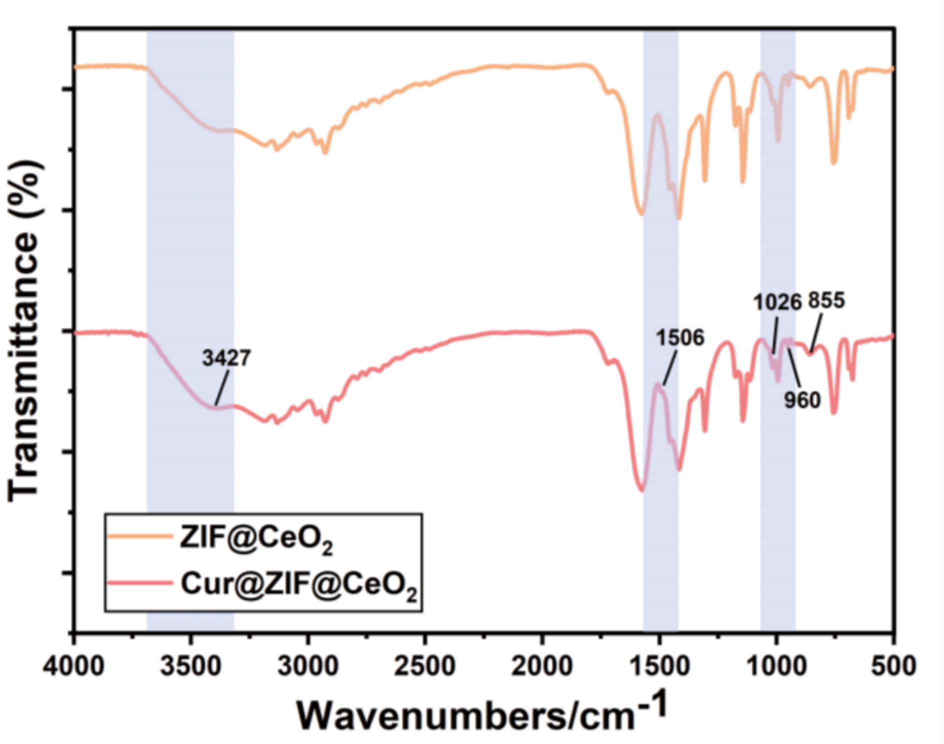

Figure S3. Fourier transforms infrared spectroscopy of ZIF@CeO_2 and CZC.

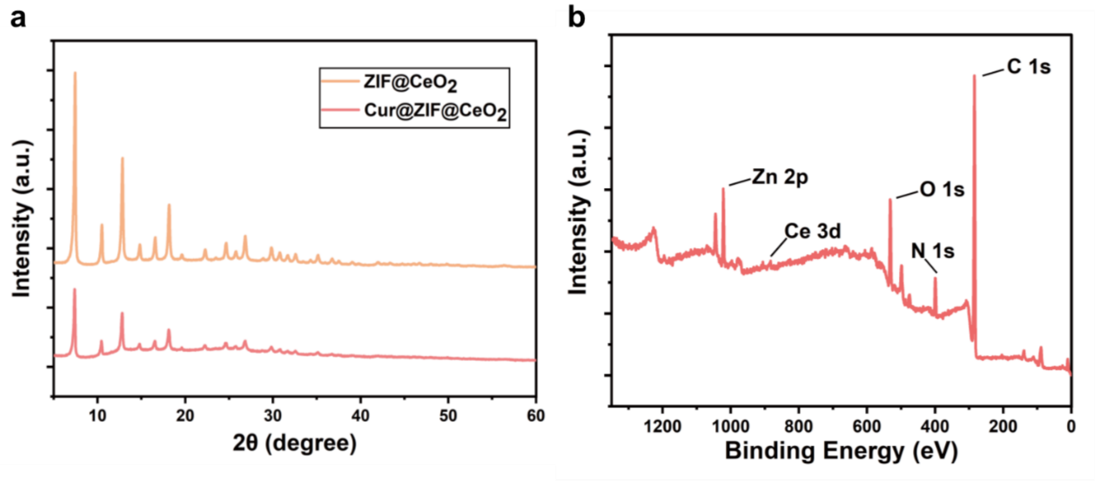

Figure S4. (a)XRD of ZIF@CeO_2 and CZC and (b)XPS survey of CZC.

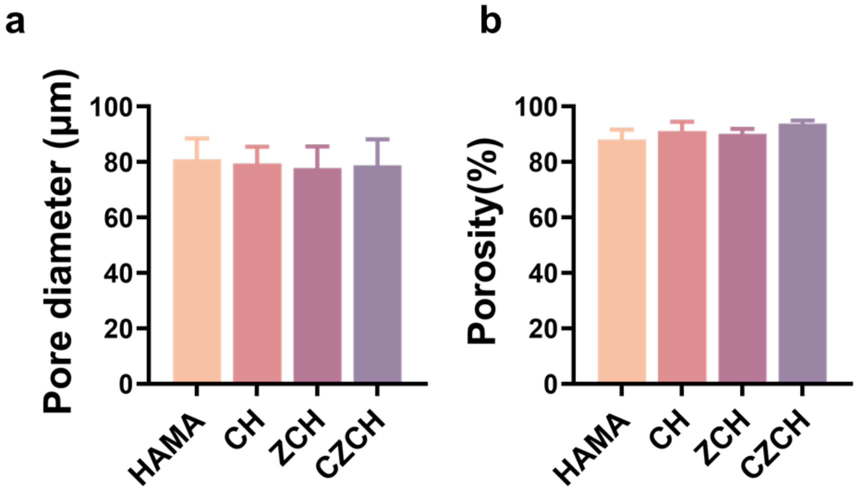

Figure S5. (a)Pore diameter and (b)porosity percentage of HAMA, CH, ZCH, and CZCH.

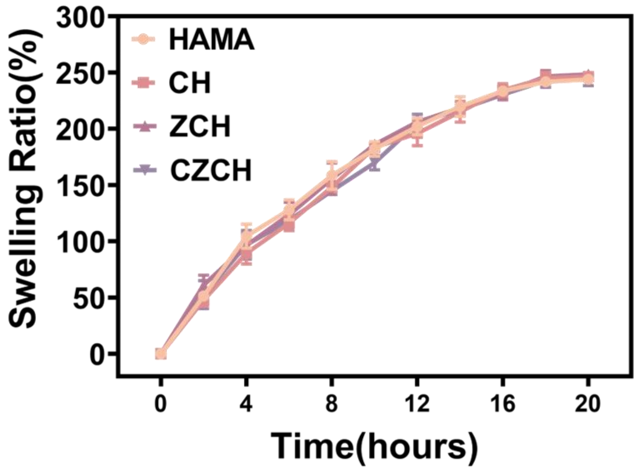

Figure S6. Swelling curves of HAMA, CH, ZCH, and CZCH in PBS solution.

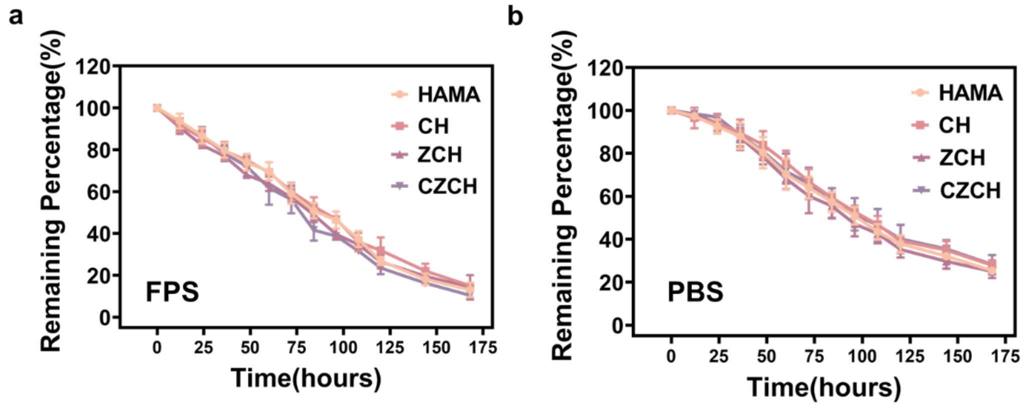

Figure S7. Degradation curves of HAMA, CH, ZCH, and CZCH in FBS and PBS solution.

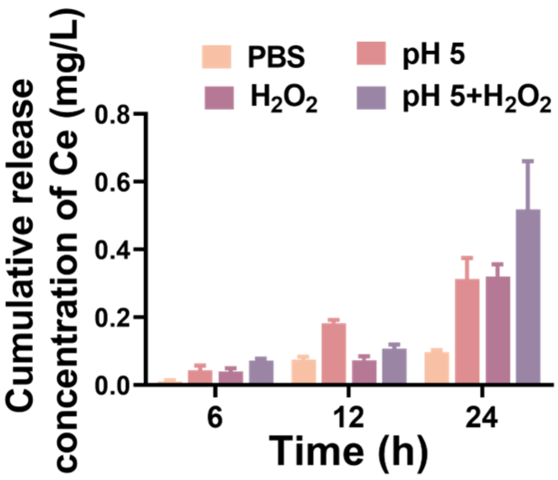

Figure S8. Cumulative Ce concentration released from CZCH in different conditioned solutions as determined by ICP-MS

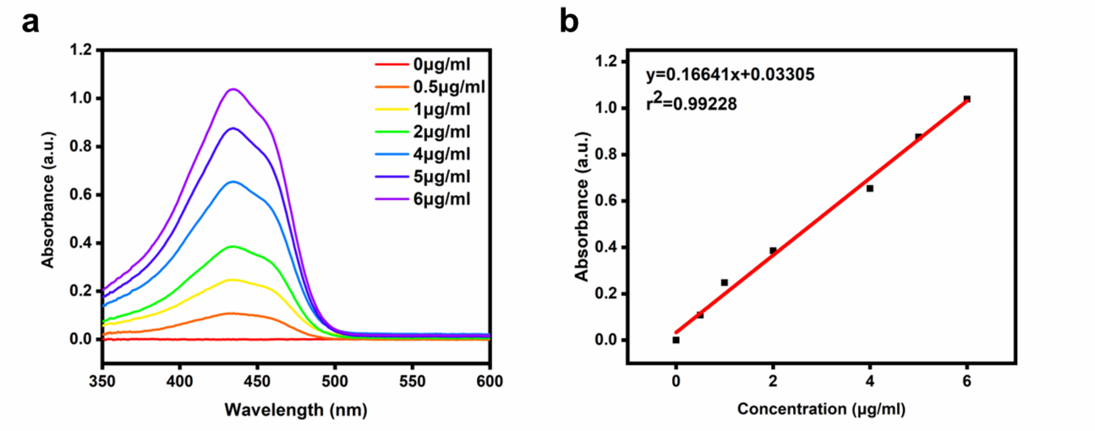

Figure S9. (a) UV-vis spectra and (b) standard curve of curcumin.

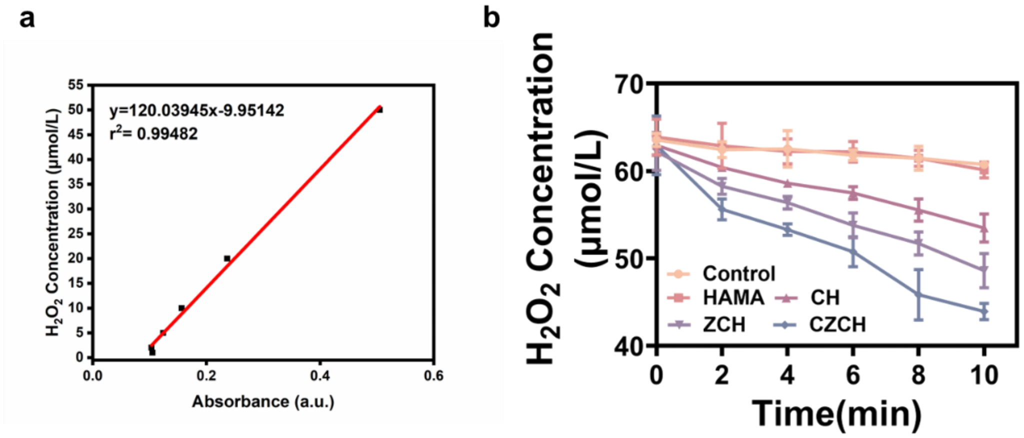

Figure S10. (a) Standard curve of H_2O_2 and (b) remaining concentration after different treatments.

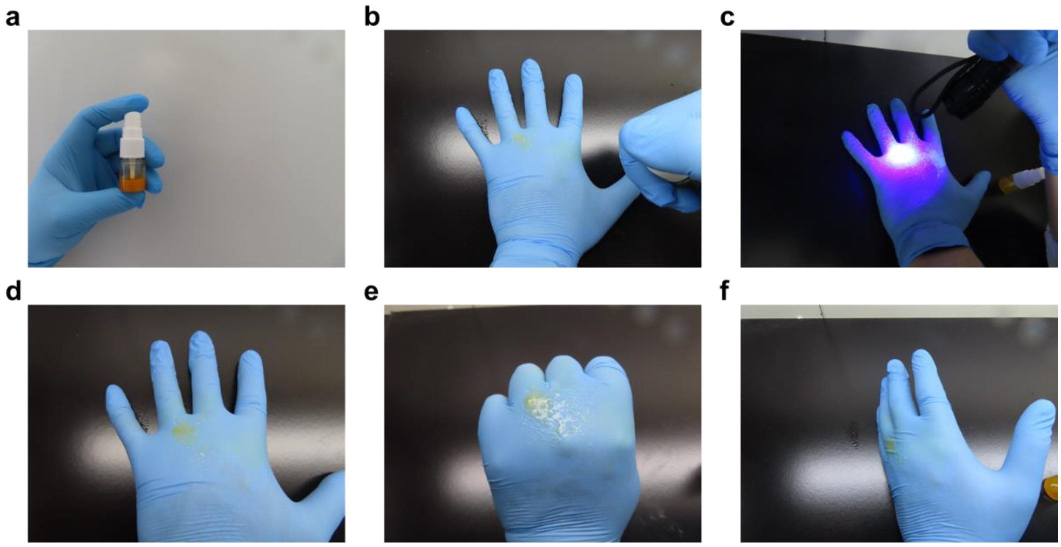

Figure S11. (a) CZCH model and (b) spray, (c) photo-crosslink, (d-f) CZCH hydrogel film in different positions.

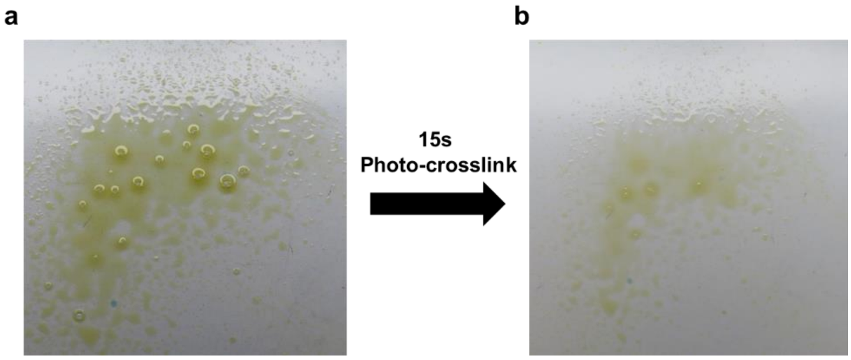

Figure S12. Hydrogel film formed by CZCH sprayed on smooth metal after 15 seconds of rapid photo-crosslinking.

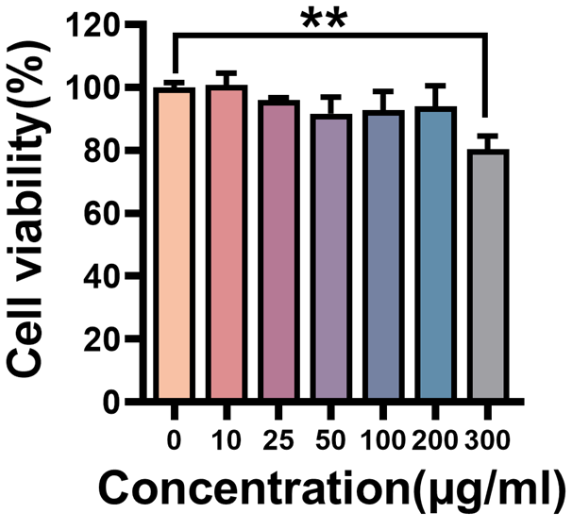

Figure S13. Relative cell viability of RAW 264.7 cells after co-incubation with different concentrations of CZCH as determined by the CCK-8 method. ** $p<0.01$.

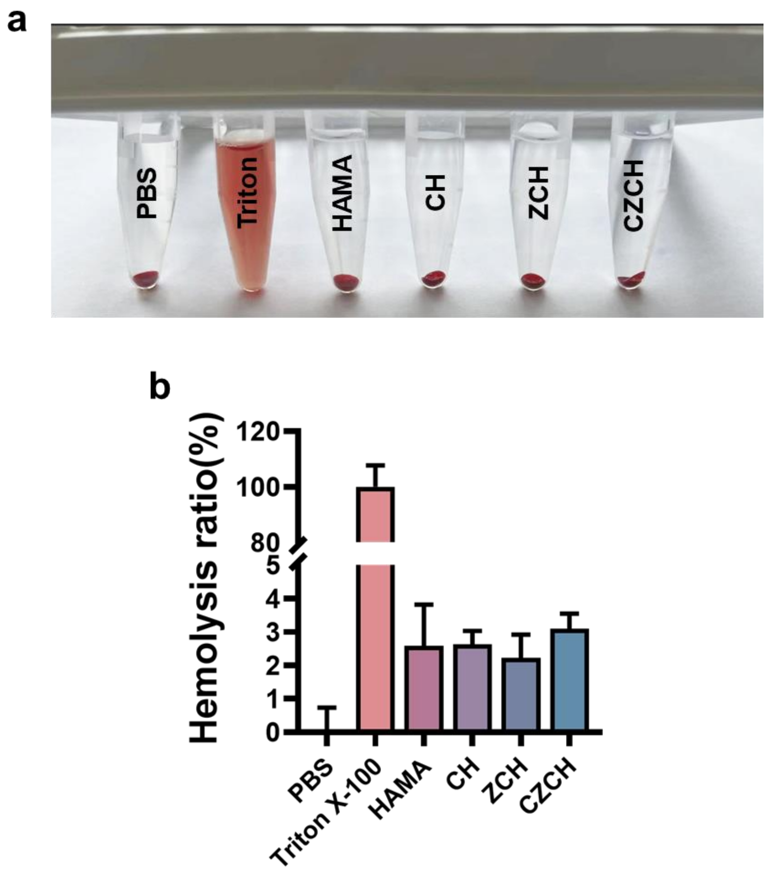

Figure S14. Hemolysis graph and quantification of hemolysis ratio of erythrocytes after co-incubation with different groups. The PBS group was set as the negative group while the Triton-X group was set as the positive control.

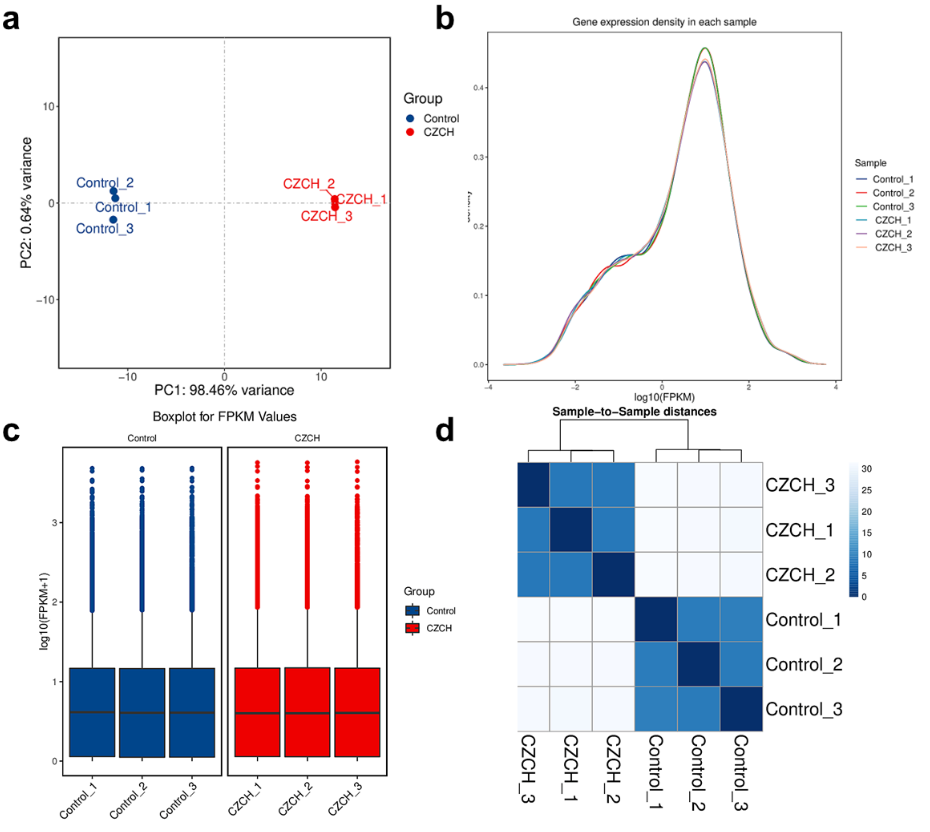

Figure S15. (a) Principal component analysis (PCA) and (b) gene expression density plots within each group, (c) FPKM box plots, and (d) sample-to-sample distance heat maps between Control and CZCH groups.

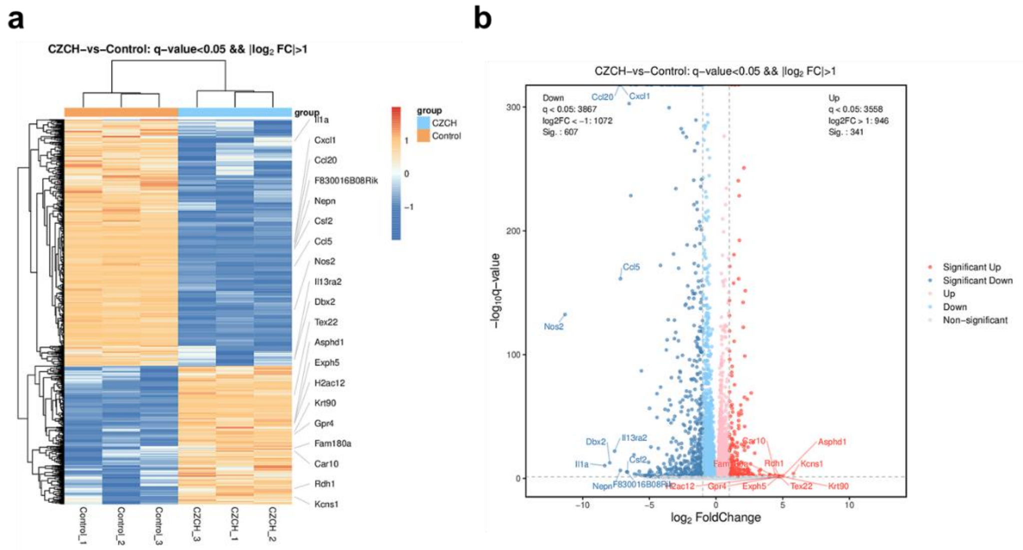

Figure S16. (a) Heat map of group differences and (b) volcano map of differential gene expression between the Control and CZCH groups.

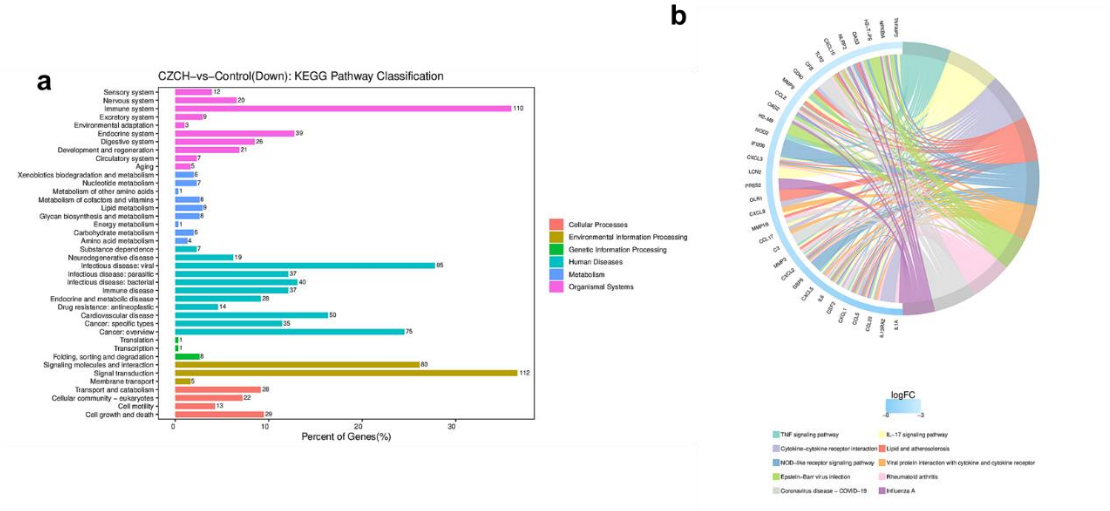

Figure S17. (a) KEGG-enriched pathway classification histogram and (b) KEGG-enriched pathway chord diagram between the Control and CZCH groups.

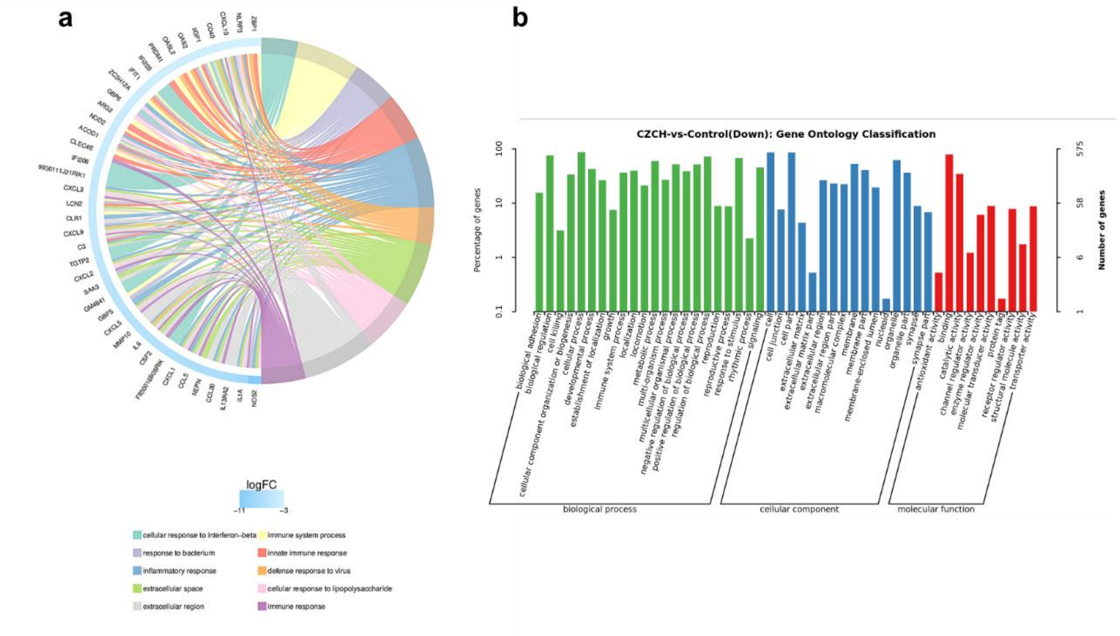

Figure S18. (a) GO-enriched pathway chord diagram and (b) KEGG-enriched pathway classification histogram between the Control and CZCH groups.

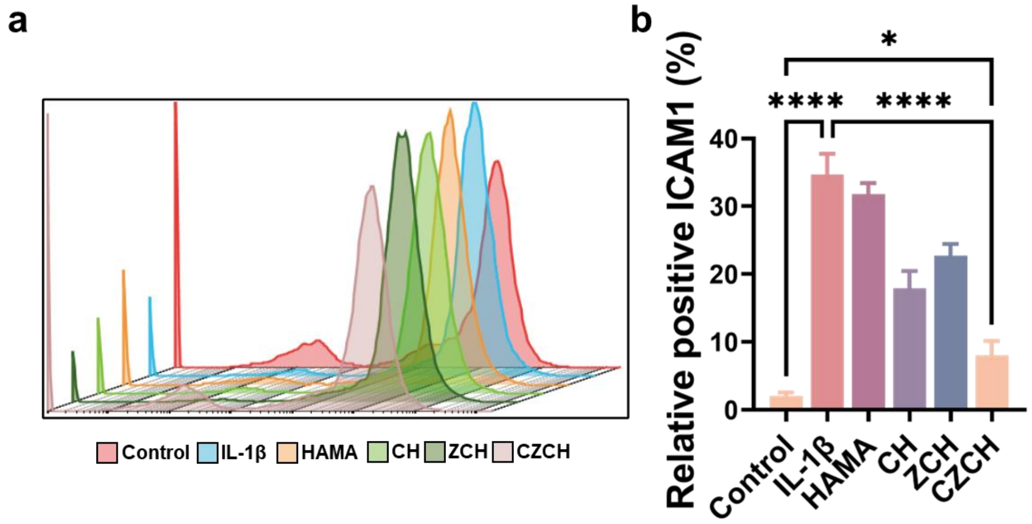

Figure S19. (a)The flow cytometry results of ICAM1 expressed by macrophages in each group and (b) the relative ratio of positive ICAM1 cell population. *p<0.05, ****p<0.0001.

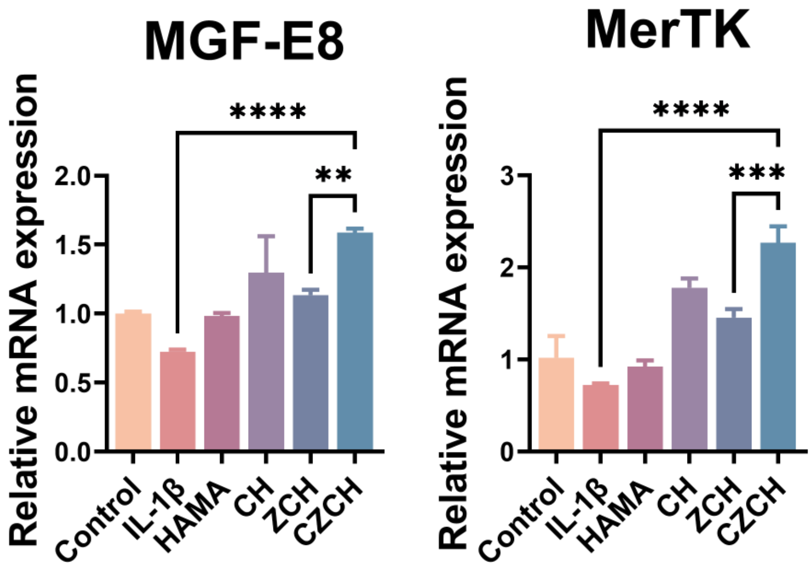

Figure S20. Relative mRNA expression of MGF-E8 and MerTK in macrophages after incubation with each condition. **p<0.01, ***p<0.001, ****p<0.0001.

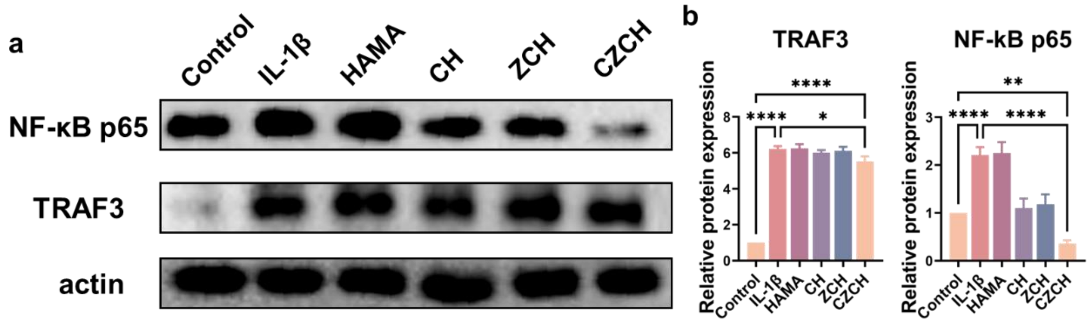

Figure S21. (a) WB band plots of NF-κB p65 and TRAF3 and (b) the relative protein quantification in tendon stem cells stimulated with hydrogel-conditioned medium pre-incubated with macrophages. β-actin was set as the internal reference protein. *p<0.05, **p<0.005, ****p<0.0001.

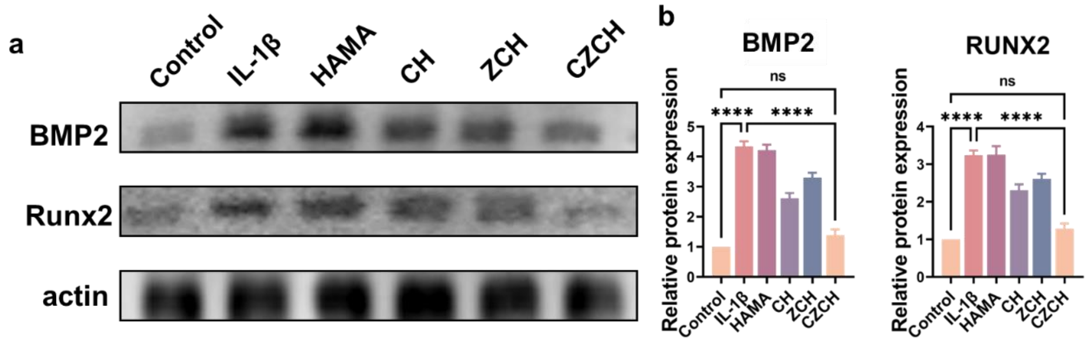

Figure S22. (a) WB band plots of BMP2 and Runx2 and (b) the relative protein quantification of tendon stem cells after stimulation with hydrogel-conditioned medium pre-incubated with macrophages. β-actin was set as the internal reference protein. ****p<0.0001, ns means no significance.

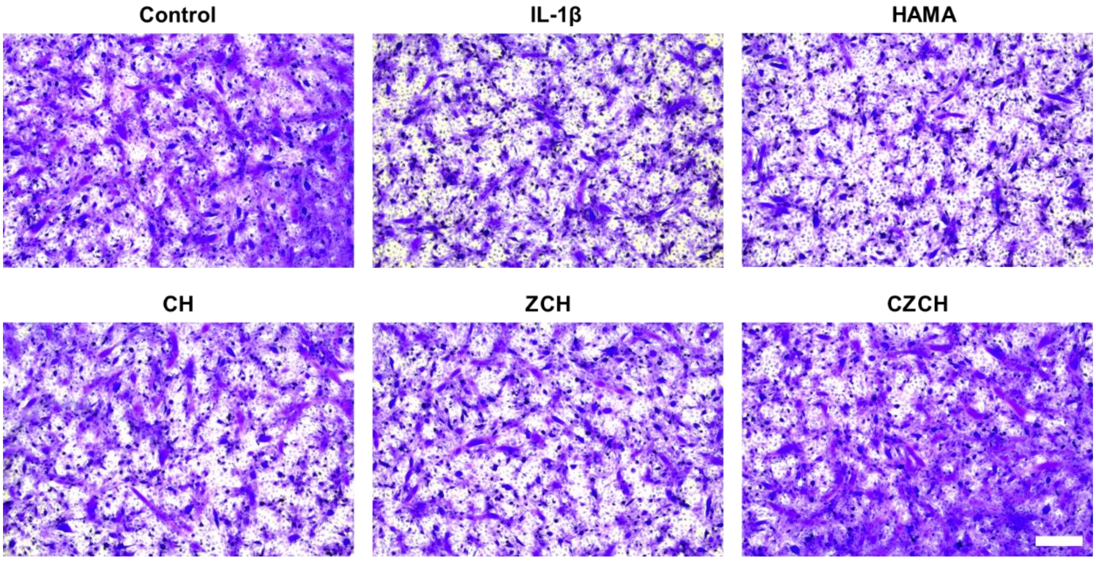

Figure S23. Crystalline violet staining plots of myogenic stem cells C2C12 stimulated by hydrogel-conditioned medium pre-incubated with macrophages. Scale bar, 250 μm.

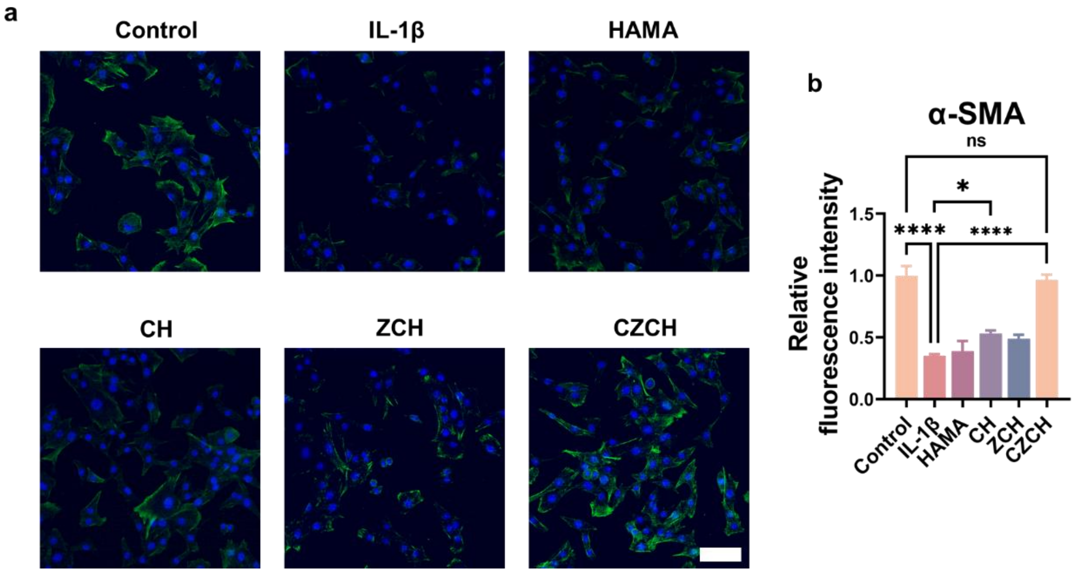

Figure S24. (a) Immunofluorescence staining of α-SMA of myogenic stem cells C2C12 stimulated by hydrogel-conditioned medium pre-incubated with macrophages and (b) the relative fluorescence quantification. *$p<0.05$, ****$p<0.0001$, ns means no significance. Scale bar, 50 μm.

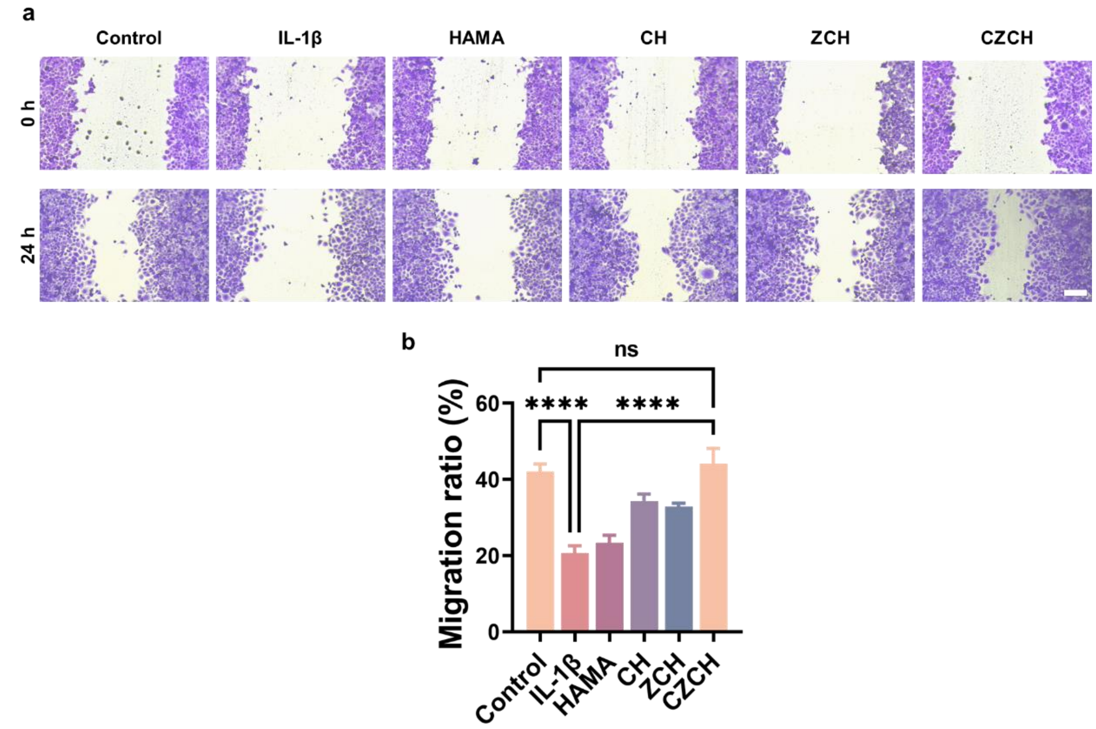

Figure S25. (a) The migration graphs and (b) quantification of migration ratios of L929 cells after treatments for 24 h with a hydrogel-conditioned medium pre-incubated with macrophages. ****p<0.0001, ns means no significance. Scale bar, 200 μm.

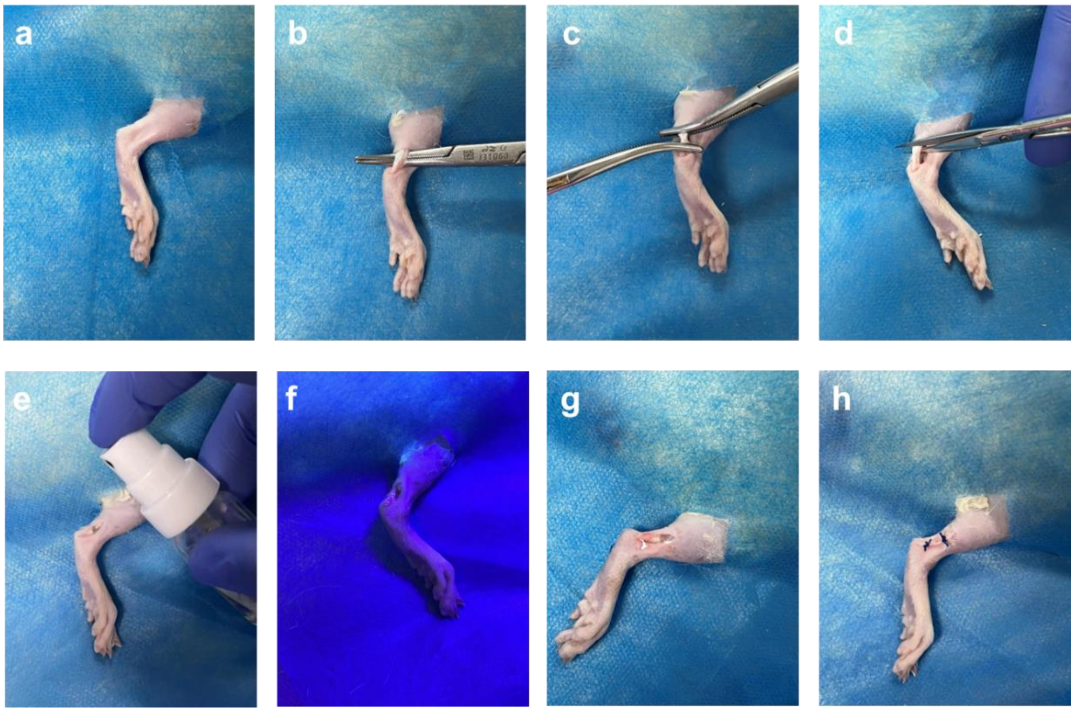

Figure S26. Schematic diagram of rat Achilles tendon THO constructs surgery with CZCH treatment. (a) Surgical position and scope of sterilization, (b) exposure of Achilles tendon, (c) clamping of Achilles tendon, (d) clipping of Achilles tendon, (e) CZCH spray treatment, (f) intraoperative rapid photo-crosslinking, (g) CZCH hydrogel film formation and (h) suturing of the wound.

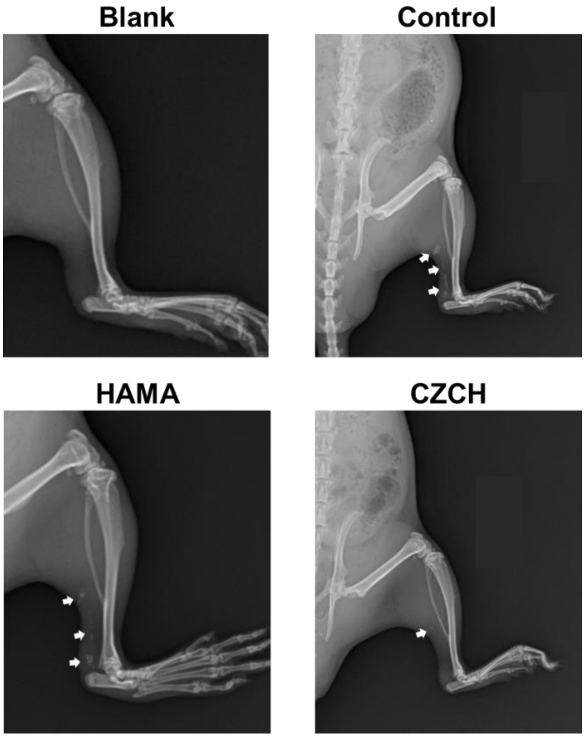

Figure S27. X-ray images of the rat Achilles tendon 14 weeks after THO construction surgery and CZCH treatment. The white arrow points to the THO formation.

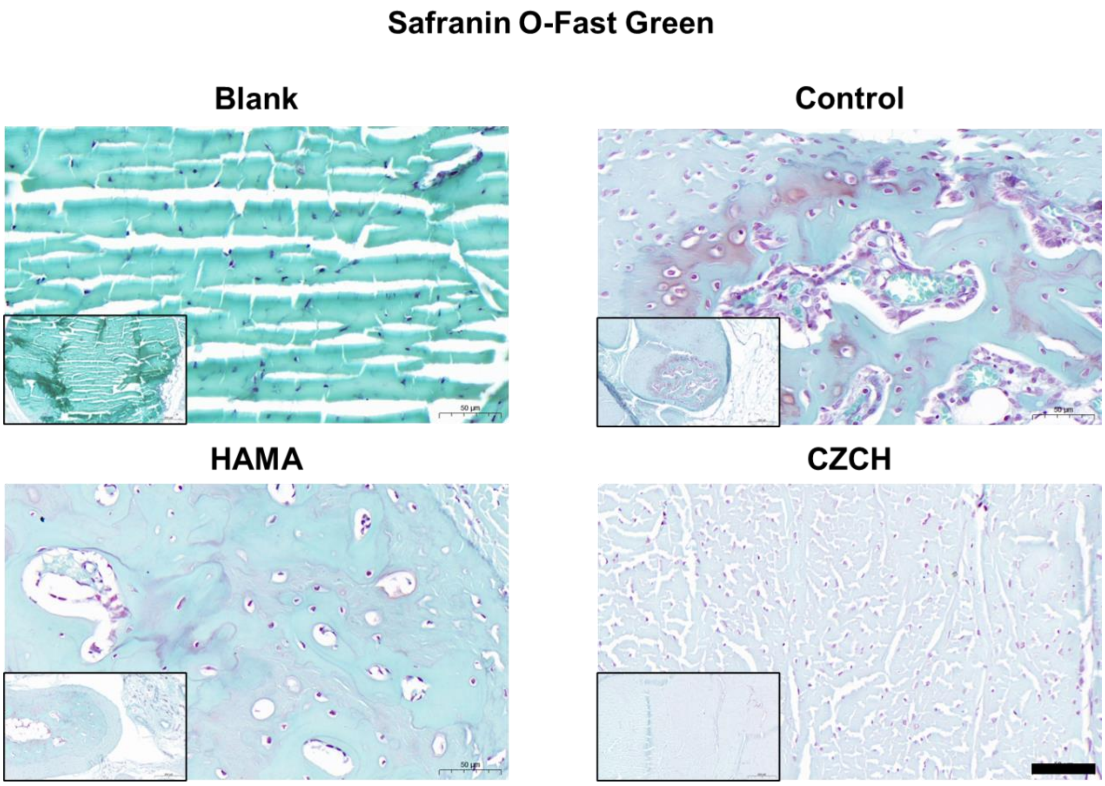

Figure S28. Images of cross-sectional sections at the Achilles tendon of rats at 14 weeks stained with safranin O-Fast green. The large image is 63x magnification and the embedded small image is 20x magnification. Scale bar, 50 μm.

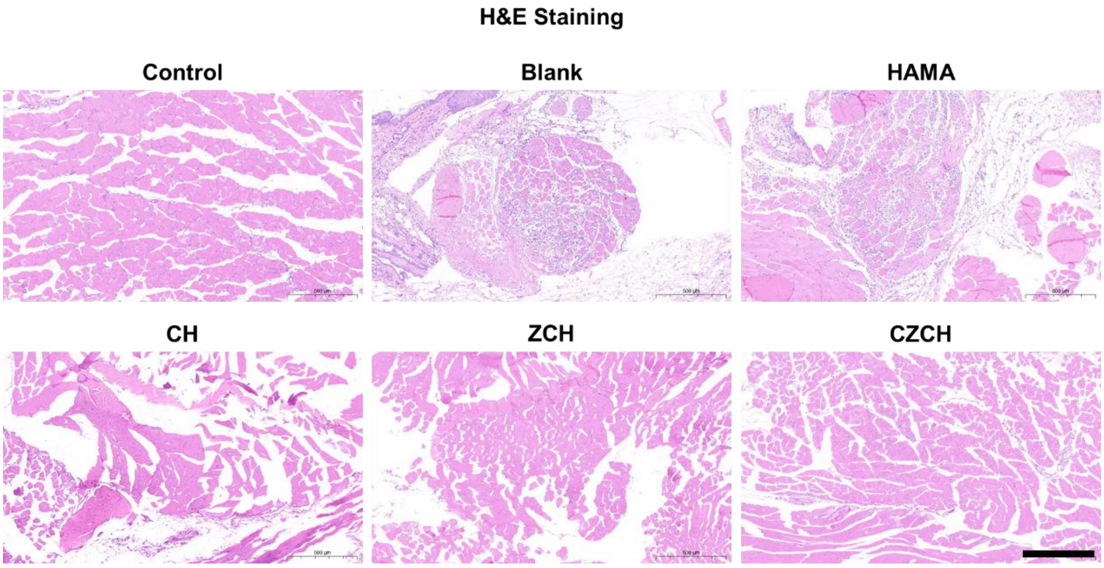

Figure S29. Images of cross-sectional sections at the Achilles tendon of the mouse on Day 5 stained with H&E. The large image is 10x magnification. Scale bar, 500 μm.

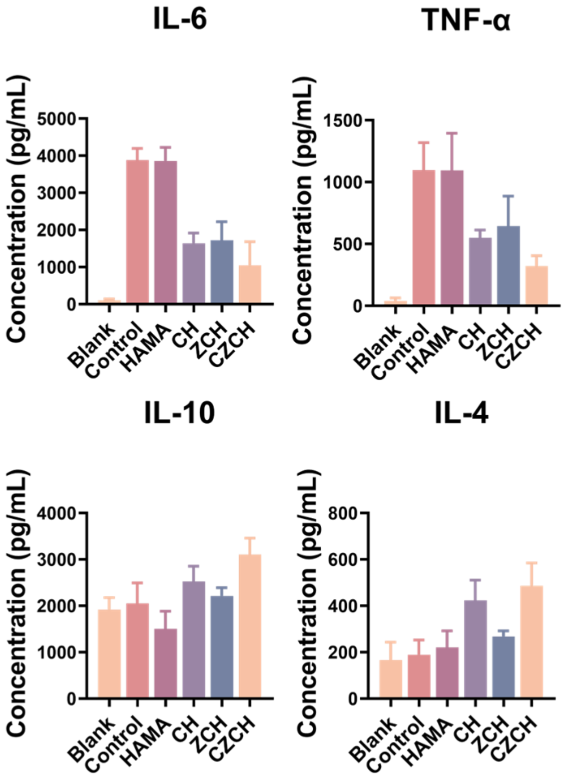

Figure S30. The cytokine concentrations in the tissue homogenate at the Achilles tendon of mice were determined by ELISA on day 5 after treatment in each group.

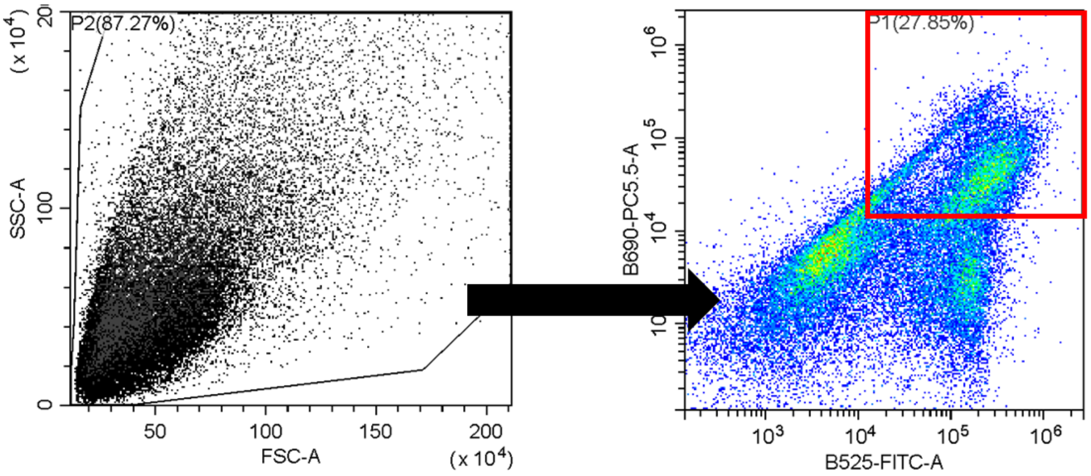

Figure S31. Representative gating strategy of macrophages in single-cell suspensions of tissue at the mouse Achilles tendon. FITC-CD11b and PC5.5-F4/80 double-positive cells (red frame) were the macrophages in the cell population for subsequent flow cytometric assays.

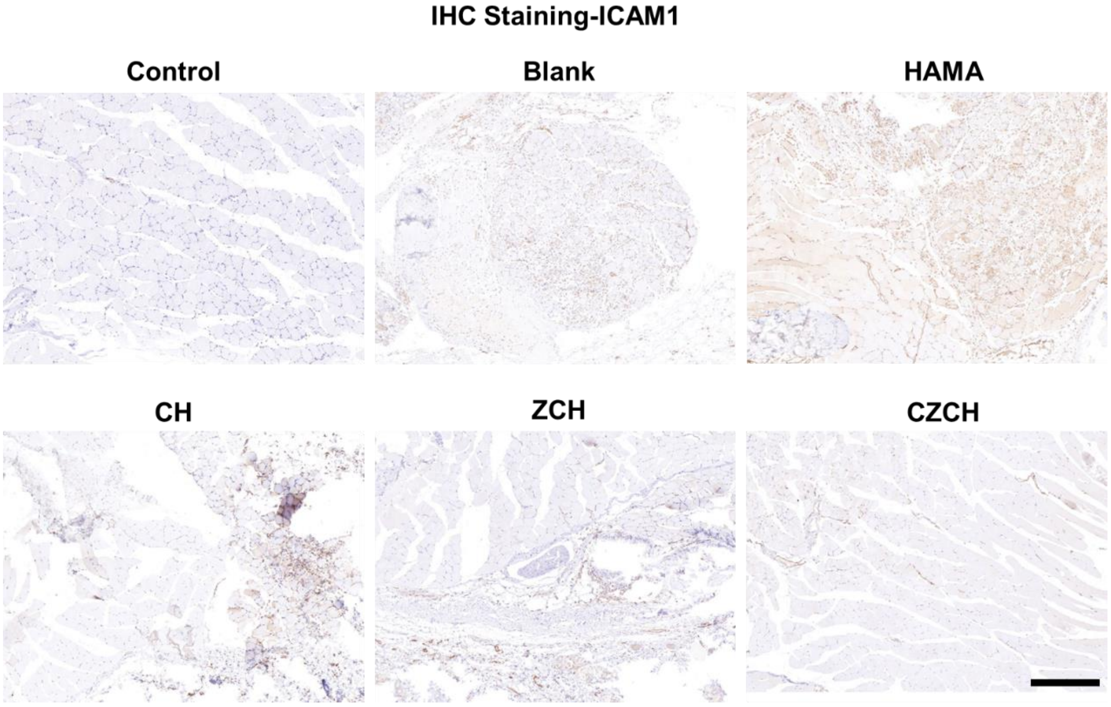

Figure S32 Immunohistochemical anti-mouse ICAM1 staining images of cross-sectional sections at the Achilles tendon of mice at day 5. The picture is 10x magnification. Scale bar, 500 μm.

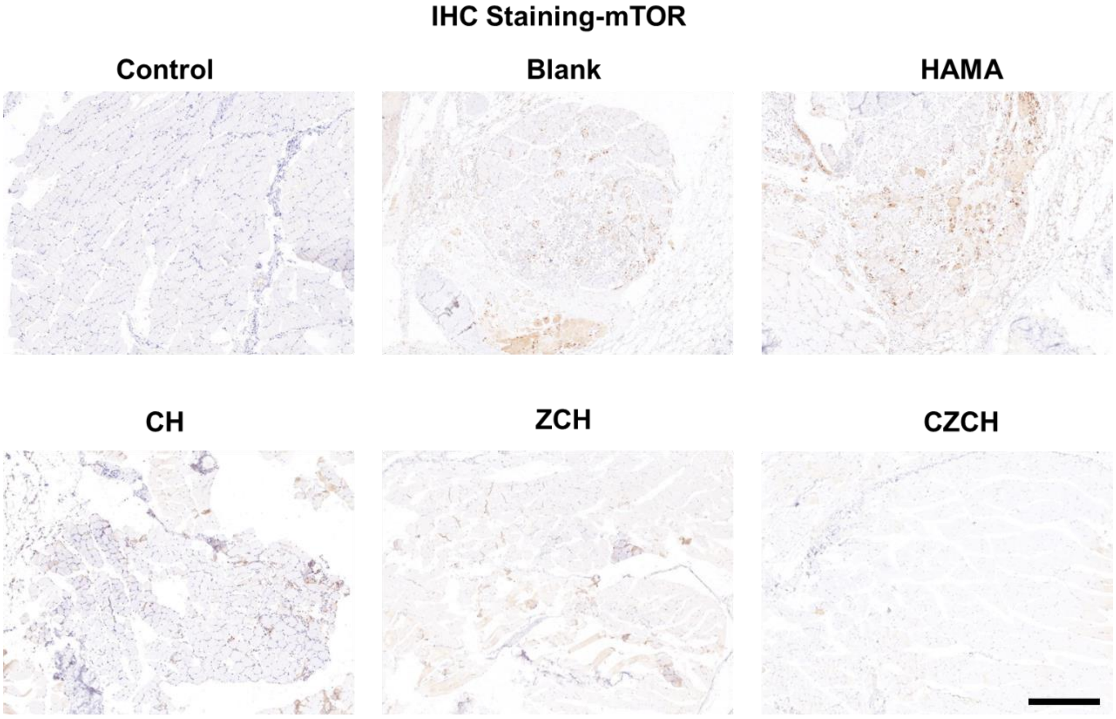

Figure S33. Immunohistochemical anti-mouse mTOR staining images of cross-sectional sections at the Achilles tendon of mice at day 5. The picture is 10x magnification. Scale bar, 500 μm.

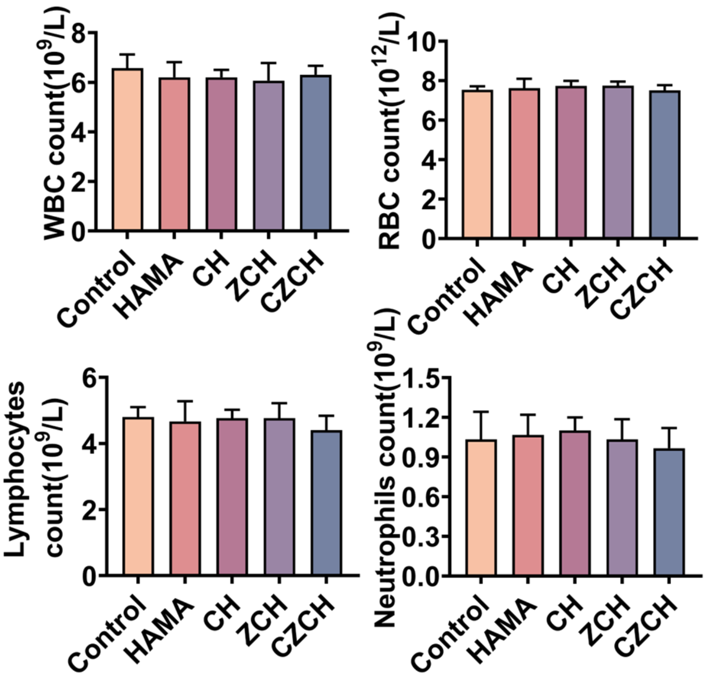

Figure S34. Routine functional indicators of peripheral blood in the mouse THO model at week 8 after treatment with different groups.

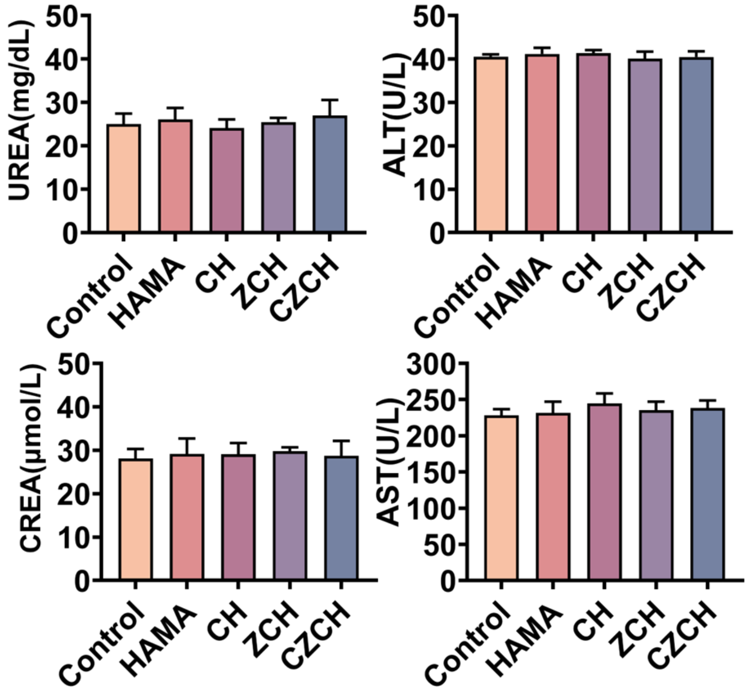

Figure S35. Peripheral blood renal and liver function indicators at week 8 after treatment with different groups in a mouse THO model.

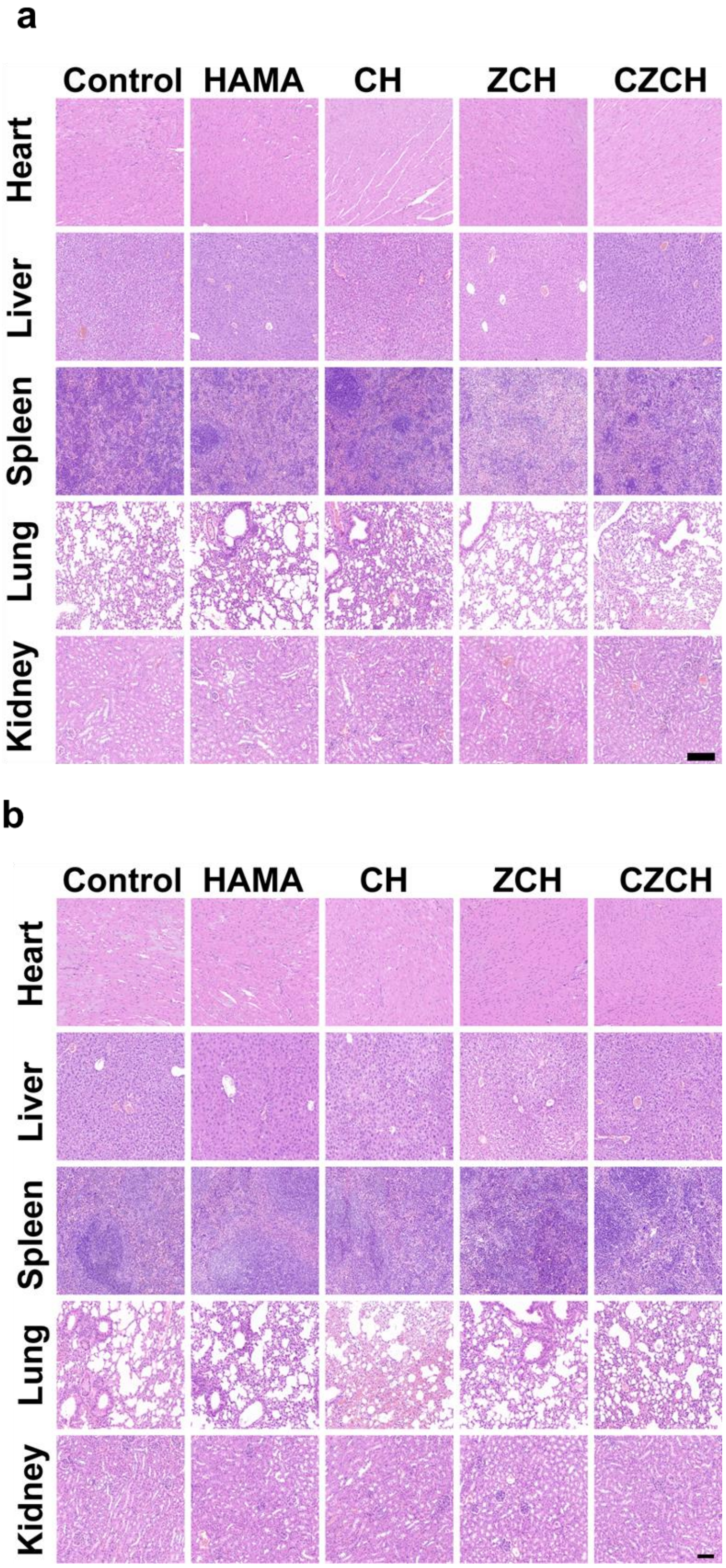

Figure S36. H&E staining of vital organs in the mouse THO model at week 8 after CZCH treatment and in the rat THO model at week 15 after CZCH treatment. Scale bar, 100 μm.
